# Neuronal Firing Sensitivity to Morphologic and Active Membrane Parameters

**DOI:** 10.1371/journal.pcbi.0040011

**Published:** 2008-01-18

**Authors:** Christina M Weaver, Susan L Wearne

**Affiliations:** 1 Laboratory of Biomathematics, Mount Sinai School of Medicine, New York, New York, United States of America; 2 Computational Neurobiology and Imaging Center, Mount Sinai School of Medicine, New York, New York, United States of America; 3 Department of Neuroscience, Mount Sinai School of Medicine, New York, New York, United States of America; UFR Biomédicale de l'Université René Descartes, France

## Abstract

Both the excitability of a neuron's membrane, driven by active ion channels, and dendritic morphology contribute to neuronal firing dynamics, but the relative importance and interactions between these features remain poorly understood. Recent modeling studies have shown that different combinations of active conductances can evoke similar firing patterns, but have neglected how morphology might contribute to homeostasis. Parameterizing the morphology of a cylindrical dendrite, we introduce a novel application of mathematical sensitivity analysis that *quantifies* how dendritic length, diameter, and surface area influence neuronal firing, and *compares* these effects directly against those of active parameters. The method was applied to a model of neurons from goldfish Area II. These neurons exhibit, and likely contribute to, persistent activity in eye velocity storage, a simple model of working memory. We introduce sensitivity landscapes, defined by local sensitivity analyses of firing rate and gain to each parameter, performed globally across the parameter space. Principal directions over which sensitivity to all parameters varied most revealed intrinsic currents that most controlled model output. We found domains where different groups of parameters had the highest sensitivities, suggesting that interactions within each group shaped firing behaviors within each specific domain. Application of our method, and its characterization of which models were sensitive to general morphologic features, will lead to advances in understanding how realistic morphology participates in functional homeostasis. Significantly, we can predict *which* active conductances, and *how many* of them, will compensate for a given age- or development-related structural change, or will offset a morphologic perturbation resulting from trauma or neurodegenerative disorder, to restore normal function. Our method can be adapted to analyze any computational model. Thus, sensitivity landscapes, and the quantitative predictions they provide, can give new insight into mechanisms of homeostasis in any biological system.

## Author Summary

Homeostasis is a process that allows a system to maintain a certain level of output over a long time, even though the inputs controlling the output are changing. Recently, studies of neurons and neuronal networks have shown that the “active” parameters that describe the movement of ions across the cell membrane contribute to homeostasis, since these parameters can be combined in different ways to maintain a specific output. There is also evidence that the physical shape (“morphology”) of the neuron may play a role in homeostasis, but this possibility has not been explored in computational models. We have developed a method that uses sensitivity analysis to evaluate how different kinds of parameters, like active and morphologic ones, affect model output. Across a multi-dimensional parameter space, we identified both local and global trends in parameter sensitivities that indicate regions where different parameters, even morphologic ones, contribute strongly to homeostasis. Significantly, the authors used sensitivities to predict *which* parameters should change, and by *how much*, to compensate for changes in another parameter to restore normal function. These predictions may prove important to neuronal aging, disease, and trauma research, but the method can be used to analyze any computational model.

## Introduction

Recent studies have demonstrated that neurons and neuronal networks are capable of functional homeostasis, maintaining specific levels of neural activity over long time scales. Although the combination of currents within individual neurons of the same class varies widely, the output of these neurons and even the network as a whole remains remarkably stable [1–7]. Interestingly, in some circumstances function is maintained despite changes in neuronal *morphology* [4]. Computational models have been used to explore processes underlying neuronal homeostasis [8], and have demonstrated that many combinations of conductance parameters across the parameter space can evoke similar firing patterns [2,3,6]. Neuronal morphology also seems to be under homeostatic control: global features, including the distribution of dendritic mass, are conserved across different classes of neurons, making it unlikely that local morphologic features, such as dendritic length and numbers of branches, are regulated independently [9,10]. Nonetheless, computational models have not yet explored how morphology might contribute to functional homeostasis.

Dendritic morphology is a critical determinant of neuronal firing dynamics and signal processing [11–16]. The influence of morphology on neuronal processing is further enhanced by active ion channels distributed throughout the dendrites [14,17–19]. Previous computational studies have identified conductance [2,3,6,20] and morphologic [11,21,22] parameters that drive general firing patterns, but have not *quantified* how these different parameters influence individual models across parameter space. Moreover, few studies have analyzed the contributions of intracellular calcium (Ca^2+^) dynamics to firing patterns [21], despite the crucial role of intracellular Ca^2+^ in shaping membrane potential, synaptic transmission, and signaling cascades [23].

Working memory, which maintains a brief mental representation of a recent event necessary for future task performance [24,25], is one function thought to exploit the computational capacity of dendrites [26,27]. Persistent neural activity, a hallmark of working memory, has been observed throughout the brain. Neurons from the pre-cerebellar nucleus Area II of goldfish exhibit, and likely contribute to, eye velocity storage [28–31], a mechanism that displays persistent activity long after termination of its eliciting stimulus. Experiments suggest that intrinsic properties of individual cells contribute to persistent activity (see Major and Tank [32] for review). We hypothesize that morphologic properties of Area II neurons partially compensate for intrinsic differences in active and passive conductances to maintain similar firing patterns throughout the nucleus [30,31].

To begin to test hypotheses like this, we introduce a novel application of mathematical sensitivity analysis [33] in which we quantify the effects of different classes of parameters, like active conductances and morphology, on model output. This quantification allows us to compare the effects of each of these parameters on models across parameter space that produce similar output, identifying mechanisms of homeostasis. Sensitivity analysis has been used widely in physics, chemical engineering, and biological signaling models to understand the relationship between model input and output [33–36], but its use within computational neuroscience has been limited [37,38]. Traditionally, sensitivity analysis is performed locally around a single optimal model, or globally reporting a general sensitivity throughout the parameter space, to quantify how each parameter contributes to model output. The large number of parameters and their nonlinear interactions render such techniques insufficient over the broad parameter space of neuronal compartment models. To synthesize these approaches, we explore sensitivity landscapes, which provide a global picture of parameter sensitivities while identifying how intrinsic properties of individual neurons influence their firing patterns. This method can be adapted easily to compare sensitivities of parameters in any computational model.

Our method is defined by three basic steps: parameterizing morphology to permit its systematic variation; evaluating the sensitivity to small perturbations of models across parameter space; and exploring sensitivity landscapes constructed from the local sensitivities to identify global trends. To simplify the presentation of our method, we use a reduced morphologic model comprising a soma and cylindrical dendrite. We later demonstrate that our sensitivity analysis method can be applied to models that include more realistic morphologies. Our analysis identified principal directions over which sensitivity magnitude, and even sign, to all parameters varied most. At the same time, we found domains across the space in which different groups of parameters, even morphologic ones, had the highest sensitivities. Together, these results reveal mechanisms that maintain homeostasis, both locally and globally across parameter space. We show how sensitivities can be used to predict compensatory tradeoffs quantitatively, between morphologic and conductance parameters that can maintain target activity levels in Area II cells. Such compensatory mechanisms exist in many systems where function is conserved despite variability in dendritic structure, synaptic input, or membrane excitability [4,39], and may offset some of the morphologic changes associated with aging and neurodegenerative disorders (reviewed by Dickstein et al. [40]). Sensitivity landscapes provide new insight into mechanisms of functional homeostasis throughout the brain, and can facilitate analysis of homeostasis in any biological system.

## Results

**Figure 1:**
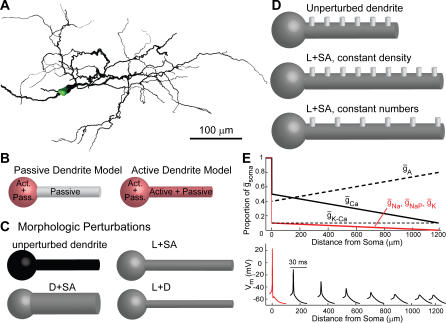
Constructing the Morphologic Model (A) Morphology of an Area II neuron traced in 3-D. The reduced morphology in (B) conserved the surface area of the soma (shown in green) and the length and surface area of the dendritic tree. The axon (truncated thick process extending from the left of the soma) was omitted. (B) Channel distributions in active and passive dendrite models. Red compartments included active channels; gray compartments included only passive ones. (C) Morphologic perturbations. Top left: unperturbed model morphology. Top right, *L* + *SA*: dendritic length *L* and surface area *SA* perturbed with dendritic diameter *D* held constant. Bottom left, *D* + *SA*: *D* and *SA* perturbed with *L* held constant. Bottom right, *L* + *D*: *L* increased and *D* decreased such that *SA* remained constant. (D) When dendritic morphology of the active dendrite was perturbed (e.g., *L* + *SA*), either the original dendritic channel density of each ion species was conserved (constant density; middle) or the number of dendritic channels was conserved (constant numbers; bottom). (E) Top, spatial distribution of active channels in the active dendrite. Dendritic channel density (as a proportion of its somatic density) either increased (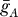 , thick dashed line), decreased (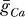 , solid black line; 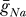 , 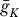 , 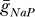 , red solid line), or remained constant (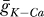 , thin dashed line). See Materials and Methods. Bottom, decremental AP backpropagation as a function of distance from the soma, for the conductance distributions shown at top. Somatic AP shown in red.

A reduced model neuron was constructed to conserve the maximal dendritic length and surface area of an Area II neuron electrophysiologically characterized in vivo [30] and reconstructed in three dimensions (3-D; Figure 1A; see Materials and Methods). The model included seven conductances, fired regular action potentials (APs) without external excitation, and exhibited a biphasic afterhyperpolarization (AHP) following the AP. The model morphology, a cylindrical soma with a constant diameter dendrite (14 compartments overall; Figure 1B), was parameterized by length (*L*), diameter (*D*), and total surface area (*SA*); somatic dimensions were held constant.

We compared the sensitivity of simulated neuronal firing patterns to two different classes of parameters: those describing active ionic membrane properties (including parameters controlling intracellular Ca^2+^ dynamics), and those describing dendritic morphology. See Table 1 for parameter classifications. Sensitivities were computed for two model types: passive dendrite “optimized models” identified by an automated parameter search as acceptable fits to target data, and Area II–like active dendrite “candidate models” identified during systematic searches of parameter space. The distinction between passive dendrite and active dendrite models is shown in Figure 1B. Sensitivity to perturbations of morphologic parameters was compared with sensitivity to active parameters.

**Table 1:**
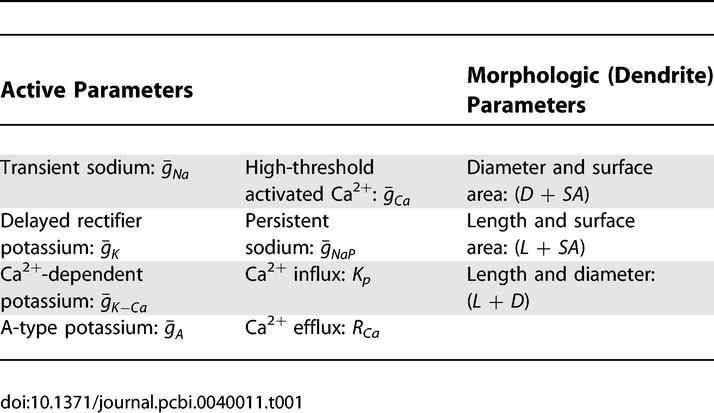
Classification of Active and Morphologic Model Parameters

Inherently associated by the relation *SA* = *π* · *L* · *D*, the three morphologic parameters *L*, *D*, and *SA* together represent only two independent parameters. To dissociate the confounded effects of *L*, *D*, and *SA*, we systematically held one parameter constant while perturbing the other two (i.e., perturb *L* and *SA*, holding *D* constant [*L* + *SA*; Figure 1C]; perturb *D* and *SA* with constant *L* [*D* + *SA*; Figure 1C]; and perturb *L* and *D* with constant *SA* [*L* + *D*; Figure 1C]). Morphologic perturbations necessarily perturb either the density or total number of active membrane channels. These confounded effects were uncoupled by performing morphologic perturbations in the active dendrite either with constant channel densities (Figure 1D, middle) or constant channel numbers (Figure 1D, bottom).

**Figure 2:**
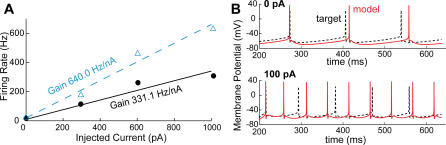
Quantifying Model Output (A) Computing firing rate gain. Black circles and solid line show firing rates and the associated fit for a model with a gain of 331.1 Hz/nA. A model with a gain of 640.0 Hz/nA is also shown (blue open triangles and dashed line). (B) Typical match of optimized model (red) against target data (black dashed line), for 0 and 100 pA injected current (top and bottom, respectively). Models were constrained to match the general AP shape, and approximate firing rates, of the synthetic target data.

Model output was quantified by two variables: spontaneous firing rate, and firing rate gain (Materials and Methods). Firing rate gain, a best-fit line to the firing rate versus current curve, provided a general measure of neuronal excitability: models with low gain were less excitable than those with high gain (compare solid black and dashed blue lines in Figure 2A). Normalized sensitivity coefficients were calculated for each model, estimating the change in each output measure to a small perturbation in each parameter (Equation 7, Materials and Methods). We explored sensitivity landscapes by evaluating normalized sensitivity coefficients across the parameter space. To demonstrate the method, we describe trends in the sensitivity landscapes of the two output variables to active and morphologic parameters for both the optimized and candidate model types. We describe trends over two generalized parameter spaces: one defined by active and passive parameters (“conductance space”), the other defined by morphologic parameters (“morphologic space”).

### Analysis of Optimized Models

Area II neurons fire spontaneously at 10.4 ± 5.8 Hz in vivo [31]. These neurons also tend to fire spontaneously in thin in vitro slices, suggesting that intrinsic currents contribute strongly to the spontaneous discharge, and exhibit a biphasic AHP (G. Gamkrelidze, personal communication). Synthetic “target” data consistent with these observations were constructed from a single compartment model [41,42] and used to constrain the simple morphologic model (Table S1; see Materials and Methods). To represent the range of firing rates observed in Area II experiments, models were optimized to reproduce the general AP shapes of the target data closely, but loosely reproduce its firing rates (Table 2; see Materials and Methods). To investigate the sensitivity of model output to morphologic parameters independent of any added conductance, the dendrite of these optimized models was made passive. The fit of a typical parameter set to the target data is shown in Figure 2B for both spontaneous firing and a 100 pA somatic current injection.

**Table 2:**
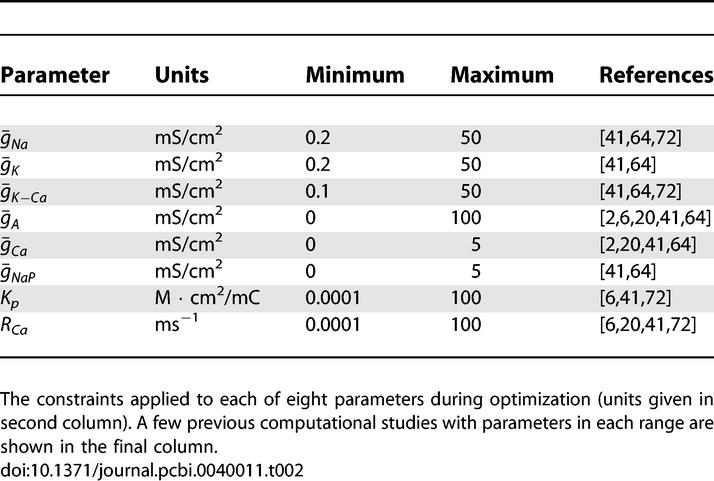
Constraints on Active Parameters Applied During Optimization

#### Spontaneous firing rate.

**Figure 3:**
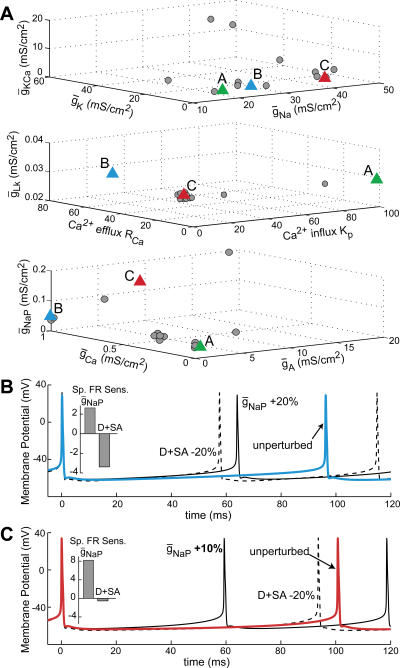
Evaluating Sensitivity for Optimized Models Across Parameter Space (A) Location in nine-dimensional parameter space of the 15 optimized passive dendrite models (gray circles and colored triangles), shown in three 3-D subspaces: [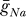 , 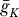 , 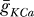 ] (top); [*K_p_*, *R_Ca_*, 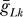 ] (middle); and [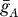 , 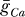 , 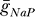 ] (bottom). Voltage traces and sensitivity coefficients of three models represented as colored triangles (A, B, and C) are compared in (B), (C), and Figure 4. (B) Comparison of morphologic (*D* + *SA*) and active parameter (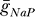 ) perturbations of the model labeled “B” (blue triangles in [A], above). Solid blue line represents the somatic voltage trace of the unperturbed model. Dashed line shows the response to a 20% decrease in *D* + *SA*; solid black line shows the response to a 20% increase in 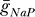 . Inset barplot shows the normalized sensitivity coefficients of spontaneous firing rate to *D* + *SA* and 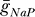 (Sp. FR Sens.): positive to 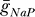 , negative and of greater magnitude to *D* + *SA*. (C) Perturbations of the model labeled “C” (red triangles in [A], above). Shown are perturbations analogous to those in (B) above, except that the solid black line shows the response to a 10% increase in 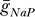 . Sensitivity to *D* + *SA* is small and negative, while sensitivity to 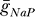 is large and positive. Note the difference in scale between the sensitivity barplot insets in (B) and (C).

The conductance space locations of 15 optimized models that matched target data are shown in Figure 3A. Nine dimensions of the conductance space (the eight optimized parameters, plus leak conductance 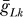 ) are illustrated as a set of three 3-D graphs. Optimized models assumed parameter values spanning the full range of conductance parameters, demonstrating that different combinations of parameters can produce similar output [2,6,20]. Interestingly, values of 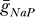 and 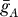 were correlated across the entire parameter space (*r* ^2^ = 0.98; Figure S1), suggesting a global compensatory interaction between enhancement (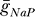 ) and reduction (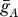 ) of excitability. There was no significant correlation between any other pair of parameters. The spontaneous firing rates of the optimized models, shown in Figure S2A, ranged from 7 to 14 Hz.

**Figure 4:**
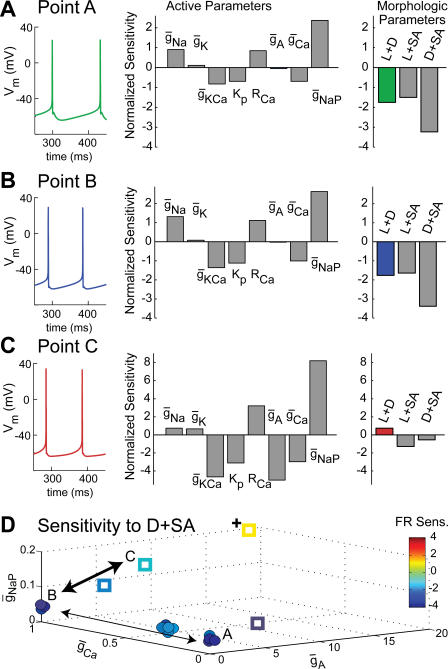
Sensitivity of Spontaneous Firing Rate to Model Parameters Varied Across Parameter Space (A–C) Somatic voltage traces and normalized sensitivity coefficients to perturbations of active (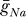 , 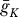 , 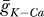 , *K_p_*, *R_Ca_*, 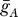 , 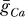 , 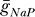 ) and morphologic (*L* + *D*, *L* + *SA*, *D* + *SA*) parameters, for the models labeled A, B, and C in Figures 3A and (D) (colored triangles). For some regions of parameter space (points A and B), the pattern of parameter sensitivities was similar. In other regions (C), the sensitivity to several parameters changed dramatically (compare colored bars in [A] and [B] versus [C]), so that the relative influence of different parameters on spontaneous firing rate was changed. (D) Firing rate sensitivity to *D* + *SA* of all optimized models, as a function of their location in the [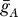 , 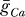 , 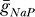 ] subspace. The points shown in (A–C) above are labeled (A, B, and C), with color indicating the magnitude and sign of firing rate sensitivity to *D* + *SA* according to the colorscale shown on the right. Arrows show the directions along which sensitivity to *D* + *SA* was relatively constant (thin arrow), and along which it changed substantially (thick arrow). Along this principal direction, even the sensitivity sign reversed (yellow circle, marked with “+”). In the 11 models shown as filled circles, firing rate sensitivity was largest to the morphologic parameter *D* + *SA* than to any active parameter. Open squares show the four models for which firing rate sensitivity was greater to at least one active parameter than to *D* + *SA*.

Sensitivity of the two model output variables to a given parameter (see Materials and Methods) depended upon a model's location in parameter space; a comparison of sensitivities at the points labeled A, B, and C in Figure 3 demonstrates this point. For the point labeled B, (blue triangles in Figure 3A), Figure 3B compares the effect on spontaneous firing rate of a 20% increase in the active parameter 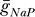 (solid black line; unperturbed model shown in solid blue), with a 20% decrease in the morphologic parameter *D* + *SA* (dashed black line). At this point, a 20% increase in 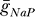 increased firing rate by 50%; hence, the sensitivity of firing rate to 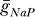 was 50% / 20% = +2.5 (sensitivities shown on inset bar plot, Figure 3B). A 20% *reduction* in *D* + *SA* also increased firing rate, this time by 67%; hence, firing rate sensitivity to *D* + *SA* was 67% / −20% = −3.35, larger in magnitude and opposite in sign to sensitivity to 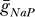 (inset bar plot, Figure 3B). In contrast, at the point labeled C (red triangles in Figure 3A), the same 20% perturbation of *D* + *SA* increased firing rate by only 8%, resulting in a much lower sensitivity value of −0.4 (*D* + *SA* reduction, dashed black line versus unperturbed model in red; sensitivity, inset bar plot, Figure 3C). The sensitivity to 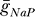 at point C (+8) was much greater than at point B, however: a 10% increase in 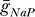 increased firing rate by more than a 20% increase at point B (solid black line and inset bar plot; compare Figure 3B and 3C).

Figure 4 shows the sensitivity of spontaneous firing rate to perturbations of each of the active and morphologic parameters (totaling 11 in all) for three models in different regions of parameter space (points labeled A, B, and C in Figures 3A and 4D). Sensitivity varied across parameter space. Sensitivity of all optimized models to perturbations of just one morphologic parameter, *D* + *SA*, is indicated by color on a scale of −4 (dark blue) to +4 (dark red) as a function of their positions in this subspace (Figure 4D). There was a clear trend of increasing sensitivity in the direction of the thick arrow in the [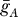 , 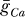 , 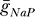 ] subspace (Figure 4D), but no trend visible by eye along other directions of the nine-dimensional parameter space. Comparing points A and B (Figure 4A and 4B), sensitivities to each parameter have the same sign and similar magnitudes, demonstrating that along some directions of parameter space, sensitivity is relatively constant (Figure 4D, thin arrow). In contrast, between points B and C (Figure 4B and 4C), sensitivity magnitude and even its sign can change (compare colored bars representing sensitivities to *L* + *D* in Figure 4A–4C). For example, from point B to point C, the sensitivity of spontaneous firing rate to *L* + *D* changed from −1.8 to 0.75. These sensitivities indicate that at point B, a 1% increase in *L* + *D* will *decrease* spontaneous firing rate by 1.8%, while at point C, a 1% increase in *L* + *D* will *increase* firing rate by a smaller amount, 0.75%. Along some directions of parameter space, sensitivity magnitude and sign changed dramatically (thick arrow in Figure 4D); a trend of increasing sensitivity along a certain direction of parameter space will hereafter be referred to as the “principal sensitivity direction.” We identified these principal sensitivity directions visually for active and morphologic parameters, and investigated how they changed when measuring different output variables.

#### Firing rate sensitivity to active and morphologic parameters.

Spontaneous firing rate was most sensitive to perturbations of 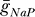 throughout the parameter space. Sensitivities to perturbations of 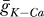 , 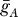 , and Ca^2+^ influx and efflux (*K_p_* and *R_Ca_*, respectively) were also high in different domains of the space. As expected, sensitivity was positive to 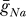 , 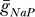 , and *R_Ca_*, and negative to 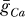 , 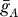 , 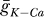 , and *K_p_*. Surprisingly, firing rate sensitivity to 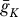 was positive, due to the prominence of the Ca^2+^-dependent potassium (K–Ca) current (Figure S3A–S3C). An increase in 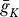 caused a deeper fast AHP; this hyperpolarized membrane potential reduced Ca^2+^ influx into the neuron. The subsequent reduction in the K–Ca current caused a smaller medium AHP. With its membrane potential closer to threshold, the model with increased 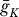 fired earlier and hence faster than the unperturbed model. Firing rate sensitivity to 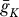 was negative in models without K–Ca current (Figure S3D).

Conceptually then, the active parameters can be grouped into those with positive sensitivity that enhance neuronal excitability (called “excitatory parameters”: 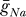 , 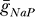 , 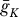 , and *R_Ca_*), those with negative sensitivity that reduce neuronal excitability (“inhibitory parameters”: 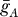 , 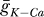 , and *K_p_*), or those with a mixed effect (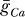 is excitatory, but activates the inhibitory K-Ca current; called “mixed”). There was a clear increase in sensitivity to all active parameters as both 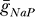 and 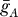 increased in the optimized models (Figure S2B). This principal sensitivity direction indicated that 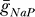 and 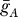 , and likely interactions between them, strongly influenced spontaneous firing rates and firing rate sensitivities.

Sensitivities of spontaneous firing rate to all morphologic parameters were consistently higher than to most active parameters. In 11 of the 15 optimized models, sensitivity to *D* + *SA* was higher than to any active parameter (filled circles, Figure 4D). Sensitivity to *L* + *SA* was negative for all optimized models, whereas sensitivity to *D* + *SA* and *L* + *D* changed sign as 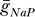 and 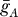 increased (Figures 4D and S2B). The principal sensitivity direction across conductance space was similar for morphologic parameters to that for active parameters (heavy arrows in Figure 4D; see also Figure S2B). These sensitivity findings can be understood from the relative contributions of somato-dendritic axial current and active membrane currents to the somatic membrane potential during interspike intervals (ISIs), and by examining how these contributions vary across parameter space (see Discussion).

#### Firing rate gain.

Some of the optimized models fired bursts or doublets in response to current injections above 250 pA. Area II neurons exhibit neither behavior. Because these models spontaneously fired single APs, they were used to evaluate trends in firing rate sensitivity, but were omitted from the analysis of gain sensitivity trends. Firing rate gains calculated from the remaining nine models ranged between 92 and 384 Hz/nA (Figure S4A).

#### Firing rate gain sensitivity to active and morphologic parameters.

Firing rate gain was generally most sensitive to perturbations of 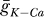 . Sensitivities to excitatory 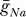 and 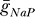 , inhibitory *K_p_*, and mixed parameter 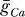 were also high in certain domains of the space. In general, gain sensitivity was positive to excitatory parameters and negative to inhibitory ones, although gain sensitivity was negative to (excitatory) 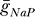 [43]. This counterintuitive sensitivity arose because increasing 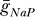 preferentially increased firing rates more at lower levels of injected current, causing an overall gain decrease. Gain sensitivity to some active parameters increased along the same principal direction as spontaneous firing rate sensitivity (e.g., 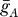 ; compare Figures S2B and S4B top right, arrow).

For most of the optimized models, firing rate gain sensitivity was high to perturbations of at least one of the morphologic parameters (Figure S4B, filled circles). Gain sensitivities varied across space, negative to *D* + *SA*, and negative to *L* + *SA* and *L* + *D* for about half the models (Figure S4B, bottom row). Gain sensitivities to *D* + *SA* and *L* + *SA* increased with 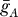 and 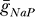 , along the same principal direction as spontaneous firing rate sensitivity (compare arrows, Figure S2B and S4B, bottom left).

To summarize, analysis of the optimized models with a passive dendrite indicated that sensitivity magnitude and sign varied throughout parameter space, changing most along the principal direction as 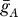 and 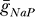 increased. Spontaneous firing rate and gain were generally more sensitive to diameter-based perturbations of dendritic morphology than to most active parameters. To characterize the shape of the sensitivity landscape and to evaluate these findings for an active dendrite, we performed systematic searches of the parameter space.

### Sensitivity Landscape Revealed by Systematic Searches of Conductance Parameter Space

Because a systematic search of sensitivities to all active and morphologic parameters of this model would be computationally prohibitive, we restricted the search to biophysically plausible ranges (Materials and Methods; Table 3) of the five dimensions over which firing rate sensitivities varied most: excitatory parameters 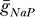 and *R_Ca_*, inhibitory parameters 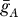 and *K_p_*, and the mixed parameter 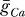 . The remaining parameters 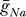 , 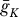 , and 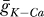 were fixed to values representative of the set of optimized models. The systematic search, conducted for the active dendrite model (Figure 1E; see Materials and Methods), is presented here. Assuming a passive dendrite yielded similar results except where noted, and these are summarized in Figures S5–S7. A summary of fixed parameters, search ranges, and grid sizes of the systematic search is given in Table 3.

**Table 3:**
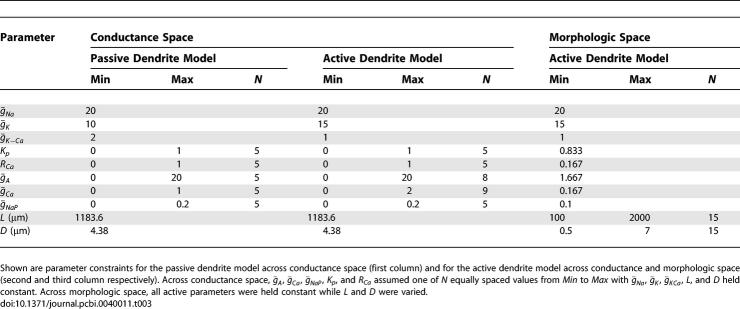
Parameter Range Constraints for Systematic Searches

#### Spontaneous firing rate.

**Figure 5:**
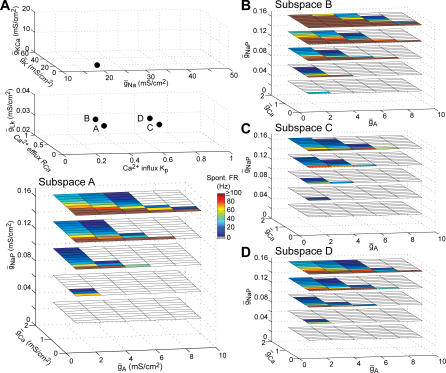
Systematic Search Across Conductance Space Revealed Continuous Variation in Baseline Spontaneous Firing Rate Throughout the systematic search, values of 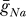 , 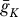 , and 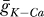 ([A], top graph) and 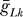 ([A], middle graph, *z*-axis) remained fixed. In each subpanel, *K_p_* and *R_Ca_* are held fixed at four different values, labeled A, B, C, and D) ([A], middle graph). A systematic search of the lower 3-D subspace [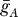 , 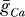 , 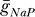 ] for each of these points is shown graphically in A, B, C, and D, respectively. Each model in the [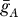 , 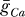 , 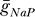 ] subspace is shown as a cell whose color represents its spontaneous firing rate, according to the colorscale on the right of subspace A. Uncolored cells were spontaneously silent models. Spontaneous firing rate depended sensitively on a balance between 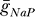 and 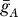 : when 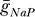 was high but 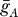 was low, rates were high, and vice versa. Firing rates also increased with *R_Ca_* (from A to B and from C to D) and inversely with *K_p_* (from A to C and from B to D).

To provide a context for the systematic search of sensitivity landscapes, we first mapped out the variation in baseline spontaneous firing rate across parameter space. Four slices through the sampled space are shown in Figure 5. The fixed parameters [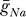 , 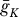 , 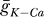 , 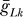 ] are marked as black circles in Figure 5A (top and middle plots). In each subpanel, *K_p_* and *R_Ca_* are held fixed at four different values, labeled (A–D) in Figure 5A, middle plot. A systematic search of the lower 3-D parameter subspace [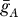 , 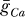 , 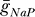 ] was performed for each of these points (Figure 5A–5D), and the results are shown graphically in Figure 5A (bottom plot), 5B, 5C, and 5D, referred to as subspaces A, B, C, and D, respectively. In each subspace, spontaneously firing models were clustered in the top left corner of the lower 3-D subspace; i.e., when 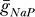 was large enough to initiate an AP, and when 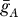 was low enough not to prevent firing. Otherwise, the neuron did not fire (uncolored cells, Figure 5). The spontaneous firing rate changed continuously, increasing with the excitatory parameter 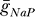 but decreasing with the mixed parameter 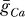 as it activated the K–Ca current. In each subspace, the number of spontaneously firing models and the peak spontaneous firing rate increased with *R_Ca_* (from point A to point B, compare Figure 5A and 5B; from point C to point D, compare Figures 5C and 5D) and decreased with *K_p_* (from point A to point C, compare Figure 5A and 5C; from point B to point D, compare Figure 5B and 5D). This finding is intuitive: small *K_p_* or large *R_Ca_* reduced intracellular Ca^2+^, which prevented a significant K–Ca current, allowing excitatory conductances to depolarize the neuron more easily.

All but 13 of the 9,000 active dendrite models fired regularly. The 13 irregular models fired doublets; 12 of them had low values of 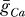 , 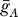 , and *R_Ca_* and high 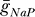 . Previous studies that have identified large regions of parameter space for which models burst spontaneously [2,20] assumed a slow intracellular Ca^2+^ removal rate, corresponding to *R_Ca_* = 0.005 ms^−1^. Larger *R_Ca_* values were used here to minimize spontaneous bursting, as Area II neurons do not exhibit this behavior.

**Figure 6:**
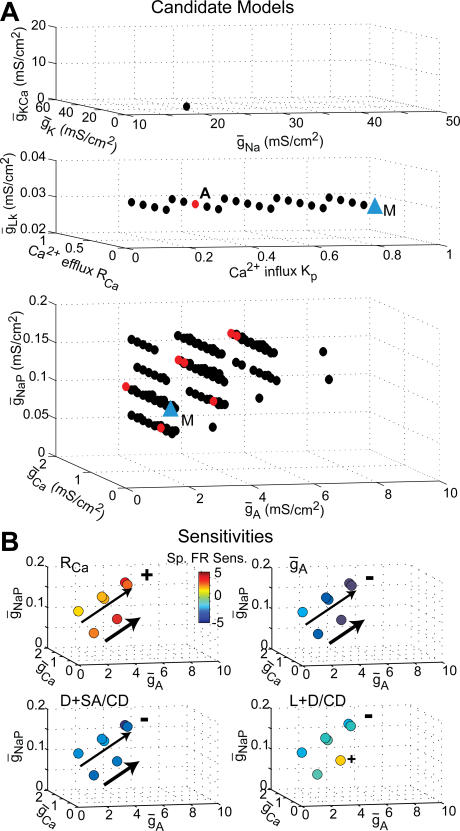
Sensitivity of Spontaneous Firing Rate Across Parameter Space (A) Colored symbols show 136 Area II–like candidate models identified during the systematic search of conductance space. Values of 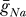 , 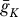 , and 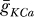 (top graph) and 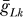 (middle graph, *z*-axis) were the same for all candidate models; locations along *K_p_* and *R_Ca_* dimensions (middle graph) and 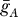 , 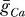 and 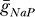 dimensions (bottom graph) varied. Some models have been shifted slightly along the 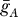 axis to aid in visualization. Red dots indicate candidate models within subspace A (Figure 5A); the blue triangle (“M”) marks the active parameter values used in the systematic search of morphologic space. (B) Sensitivity of spontaneous firing rate to parameter perturbations of the subspace A candidate models as a function of their location in the lower [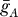 , 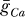 , 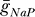 ] subspace. Color indicates sensitivity magnitude and sign according to the colorscale shown at top left. Shown are sensitivities to active parameters *R_Ca_* and 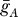 (top left and right, respectively) and to morphologic parameters *D* + *SA* and *L* + *D* with constant channel densities (“CD”; bottom left and right). Arrows indicate the principal sensitivity direction across the space.

The shape of the sensitivity landscape depended both upon location in parameter space and baseline firing rate. To analyze sensitivity trends independent of baseline firing rate effects, we focused on Area II–like “candidate models” firing spontaneously from 7–13 Hz; the gain of these models was unconstrained (see Materials and Methods). Figure 6A shows 136 candidate models identified during the systematic search. With four dimensions [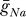 , 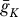 , 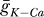 , and 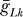 ] held fixed, these candidate models were connected across the remaining five-dimensional search space, forming a wedge in the [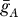 , 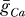 , 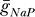 ] subspace (Figure 6A).

#### Firing rate sensitivity to active and morphologic parameters.

Figure 6B compares trends in the sensitivity of spontaneous firing rate to perturbations of two active parameters (excitatory *R_Ca_* and inhibitory 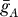 ; top row Figure 6B) and two morphologic parameters (*D* + *SA* and *L* + *D*; bottom row, Figure 6B) in the candidate models. Space limitations permit us to show only the sensitivities for models within subspace A; however, results were similar for candidate models throughout the space.

Throughout the space, firing rate was most sensitive to perturbations of 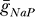 , with sensitivities to excitatory *R_Ca_* and inhibitory 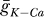 and 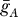 also high in different domains. Similar to the optimized models, global sensitivity was positive to perturbations of excitatory parameters and negative to perturbations of mixed and inhibitory parameters. The arrows in the top row of Figure 6B show the principal sensitivity directions to perturbations of *R_Ca_* (positive sign) and 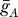 (negative sign); these trends were representative of almost all active parameters. Sensitivities increased for lower values of 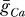 (compare thin versus thick arrows in Figure 6B) and as *R_Ca_* increased (Figure S5A, left and right columns).

Firing rate sensitivity to morphologic parameters *D* + *SA* and *L* + *D* with dendritic channel densities held constant (“constant density” [CD]) was higher than to most active parameters, among candidate models with smaller *R_Ca_* values (Figure S5A, filled circles). For these models, sensitivity to only 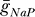 and sometimes 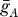 and 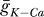 was higher than sensitivity to morphologic parameters. Sensitivities to *L* + *SA / CD* and *D* + *SA* / *CD* were negative across the parameter space: firing rate decreased as length or diameter increased, and changed sign to *L* + *D* / *CD* (Figure 6B, bottom row). Sensitivities to *L* + *SA* / *CD* and *D* + *SA / CD* increased along the same principal direction as for the active parameters (arrows, Figure 6B, bottom row).

When dendritic channel numbers were held constant (“constant numbers” [CN]), sensitivities to perturbations of *D* + *SA* / *CN* and *L* + *SA* / *CN* increased along the same principal direction as the corresponding CD perturbations, but were opposite in sign—firing rate *increased* with *D* + *SA*—and of lower magnitude (Figure S6A). Even so, when *R_Ca_* was low, sensitivity to perturbations of *D* + *SA* / *CN* was higher than to all active parameters except 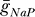 and sometimes 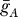 and 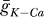 .

The relationship between CD and CN morphologic perturbations depended on the dendritic channel distributions and can be understood in terms of perturbing electrical load at the soma. Intuitively, an increase in dendritic surface area should increase the electrical load on the soma, decreasing firing rate, as occurred with CD morphologic perturbations (Figure S6A, right column). Morphologic perturbations that increase surface area holding channel numbers constant result in lower dendritic channel densities. With the predominance of inhibitory over excitatory channels in our active dendrite (see Figure 1E), increasing the surface area of the dendrite by an *L* + *SA* / *CN* or *D* + *SA* / *CN* perturbation decreased the relative density of those inhibitory channels. Because this perturbation reduced the electrical load on the soma, it *increased* the firing rate of most candidate models (Figure S6A, left column). To illustrate this point, we recomputed firing rate sensitivities after redistributing dendritic channels uniformly so that inhibition no longer dominated (Figure S6B). Firing rate sensitivity to CN morphologic perturbations that increased *SA* and hence electrical load were now negative, as expected (Figure S6B).

#### Firing rate gain.

**Figure 7:**
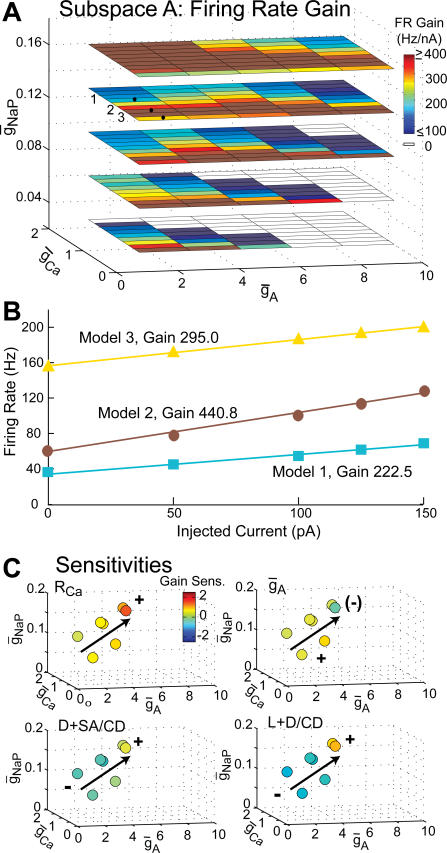
Sensitivity of Firing Rate Gain Across Conductance Space (A) Firing rate gain for models within subspace A, according to the colorscale at top right. Uncolored cells did not fire under any of the injected currents. Gain sometimes varied nonmonotonically with 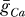 ; compare models labeled 1, 2, and 3. (B) Firing rate versus injected current with fitted gain slopes for Models 1, 2, and 3 shown in (A). Models with low 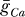 had high spontaneous firing rates but intermediate gain (Model 3, yellow triangles); gain increased for intermediate 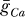 (Model 2, dark red circles), then decreased for high 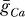 (Model 1, blue squares). (C) Sensitivity of firing rate gain to active parameters *R_Ca_* and 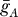 (top left and right) and constant density morphologic perturbations *D* + *SA* / *CD* and *L* + *D* / *CD* (bottom left and right) according to the colorscale at top left, for candidate models within subspace A. Arrows indicate the principal direction of global sensitivity trends across the space, which were similar for perturbations of active and morphologic parameters. The sign of sensitivity (marked by “+” and “−”) often changed along this principal direction.

The baseline firing rate gain of all active dendrite models within subspace A is illustrated as a colormap in Figure 7A. Representative gains, computed as the slopes of firing rate versus injected current, for the three models labeled (1, 2, and 3) in Figure 7A are shown in Figure 7B. Regions of lowest spontaneous firing rate and lowest gain coincided (Figure 7A and 7B, Model 1); the same was generally true for high spontaneous firing rates and gain (Figure 7A and 7B, Model 2; compare Figures 5A and 7A). The only exceptions to these observations were models with high 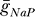 but low 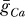 (Figure 7A and 7B, Model 3). Baseline firing rates in these models were high and nearly saturated. Since injecting current had little additional effect, these models had intermediate gain values. In a subset of the candidate models, we compared gains computed from injected somatic current against gains computed from dendritic synaptic excitation. The high correlation between these measures (*r* ^2^ = 0.992, *n* = 19; see Materials and Methods) and the linearity of each model's firing rate response to injected current (Figure 7B) demonstrated that the gain was a good measure of the firing activity over a range of inputs.

#### Firing rate gain sensitivity to active and morphologic parameters.

Among candidate models, the sensitivity of firing rate gain to perturbations of 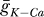 was highest throughout the space; in different domains of space, sensitivities to excitatory parameters *R_Ca_*, 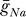 , and 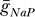 and inhibitory parameter 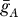 were also high. Figure 7C compares firing rate gain sensitivity to two active parameters (*R_Ca_* and 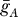 ; top row) and two morphologic parameters (*D* + *SA* and *L* + *D*; bottom row) for candidate models within subspace A. For most active parameters, sensitivities of firing rate and gain had the same sign. Gain sensitivities tended to be smaller than spontaneous firing rate sensitivities, but increased along the same principal directions (arrows, top row of Figure 7C).

As with spontaneous firing rate, gain was more sensitive to morphologic parameters *D* + *SA* / *CD* and *L* + *D* / *CD* than to most active parameters for candidate models with smaller *R_Ca_* values. Only sensitivity to 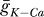 , *R_Ca_*, and sometimes 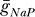 was higher in those models. Gain sensitivity to all morphologic perturbations reversed sign along the principal direction of sensitivity increase to active parameters (arrows, bottom row of Figure 7C).

Sensitivity of gain was higher to *D* + *SA* but lower to *L* + *SA* when channel numbers, rather than densities, were held constant. Otherwise, trends of sensitivity magnitude and sign were similar to those of CD morphologic perturbations. Thus, the overall effect of these morphologic perturbations on *gain* was independent of the channel density or numbers, even though CD and CN perturbations had opposite effects on *firing rates* at each level of applied current.

Gain was much lower across conductance space for the *passive* dendrite model than for the active dendrite, and decreased only slightly as 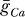 increased (Figure S7A and S7B). These differences in baseline gain caused a divergence in gain sensitivity trends between the active and passive dendrite models: gain sensitivities varied only slightly across conductance space in the passive dendrite model (note difference in sensitivity scale from Figures 7C and 7D to S7C and S7D). For the passive and active dendrite models, gain was highly sensitive to the same parameters, but the principal sensitivity directions differed (compare arrows in Figures 7C and 7D and S7C and S7D).

To evaluate the robustness of these sensitivity trends over a range of dendritic morphologies, and to examine the nature of sensitivity landscapes across morphologic space, we performed a second systematic search over the space defined by morphologic features.

### Sensitivity Landscapes Across Morphologic Space

We explored the sensitivity landscape across morphologic parameter space by systematically varying *L* and *D* while holding active and passive parameters fixed. Active parameters from the active dendrite candidate model across conductance space with lowest firing rate gain were used for the search (model marked by blue triangle labeled “M” in Figure 6A; see Materials and Methods). The search was performed twice, holding either active channel densities constant (“CD morphologic space”) or overall active channel numbers constant (“CN morphologic space”) as morphology varied (Figure 1C). Table 3 summarizes the search conditions.

#### Spontaneous firing rate across CD morphologic space.

**Figure 8:**
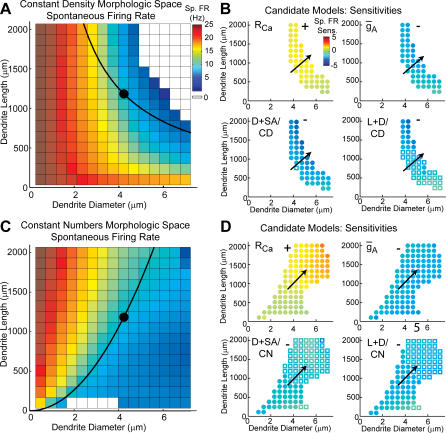
Spontaneous Firing Rate Sensitivity across Constant Density and Constant Numbers Morphologic Spaces (A) Baseline spontaneous firing rate represented as a colormap across CD morphologic space (colorscale 0–25 Hz; top right). Black dot marks the original morphology used for the conductance space searches. Thick black curve indicates all models matching the original *SA*. (B) Spontaneous firing rate sensitivities to perturbations of active parameters *R_Ca_* and 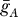 (top row), and morphologic parameters *D* + *SA* / *CD* and *L* + *D* / *CD* (bottom row) of candidate models across the space. Arrows indicate the principal sensitivity trend to each parameter. In the bottom left of (B), filled circles indicate models for which sensitivity to *D* + *SA* / *CD* was greater than sensitivity to all active parameters except 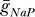 . In the model shown as an open square, sensitivity to two or more active parameters was greater than sensitivity to *D* + *SA* / *CD*. In the bottom right of (B), filled circles and open squares likewise compare the sensitivity to *L* + *D* / *CD* and sensitivities to active parameters. Analogous colormaps across CN morphologic space are shown for (C) baseline spontaneous firing rate and (D) spontaneous firing rate sensitivity to parameter perturbations among candidate models. The black curve in (C) indicates all models matching the original axial resistance (see Materials and Methods). In the bottom row of (D), filled circles show models for which sensitivity to either *D* + *SA* / *CN* or *L* + *D* / *CN* was greater than sensitivities to all active parameters except 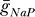 and 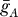 . In models shown as open squares, sensitivity to three or more active parameters was greater than sensitivity to either *D* + *SA / CN* or *L* + *D* / *CN*.

Figure 8A shows the spontaneous firing rate of models across CD morphologic space. A small, connected set of models did not fire spontaneously (uncolored cells); the others fired regularly at rates up to 25 Hz. Firing rate varied less across this morphologic space than across conductance space because active parameter values in the soma, the site of AP initiation, were held fixed along each dimension of the space. Firing rates varied continuously over the sampled points, and inversely with *D*. Models with similar firing rates lay in colored bands of vertical stripes over the lower half of the *D* range, and in approximately hyperbolic shaped bands over the upper half of the *D* range (Figure 8A). These observations revealed an interaction between *L* and *D*, largely driven by *D* over the lower half of the *D* range, and by *SA* as a whole (Figure 8A; black curve indicates constant *SA* ∝ *L* × *D *matching the original morphology). Increasing *SA* increased both the electrical load on the soma and also the number of active channels (dominated by inhibitory 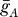 ), resulting in lower firing rates. Of the 225 models analyzed across this region of morphologic space, 47 Area II–like candidate models were identified, shown as colored symbols in the subpanels of Figure 8B.

#### Firing rate sensitivity across CD morphologic space.

The signs and relative magnitudes of firing rate sensitivities to active parameter perturbations were the same across morphologic space, as those across conductance space described above. Figure 8B compares spontaneous firing rate sensitivities to perturbations of active parameters *R_Ca_* and 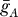 (top row) and to morphologic parameters *D* + *SA* and *L* + *D* (bottom row) for candidate models. The principal sensitivity direction to all active parameters followed increasing *SA* and decreasing baseline firing rate; as an example consider sensitivities to *R_Ca_* and 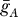 (Figure 8B, top row, arrows).

Sensitivity to perturbations of the morphologic parameter *D* + *SA* / *CD* for candidate models was greater than to all active parameters except 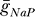 ; the same was true of *L* + *D* / *CD* for candidate models with large *L* (filled circles, Figure 8B, bottom row). Sensitivities were negative to all morphologic perturbations. The principal sensitivity direction to morphologic parameters was the same as to active parameters, increasing with *SA* and decreasing with baseline firing rate (arrows, Figure 8B, bottom row).

#### Spontaneous firing rate across CN morphologic space.

Figure 8C shows the spontaneous firing rate of models across CN morphologic space. Firing rate varied continuously up to 25 Hz and inversely with *D*. Holding CNs constant reduced the dependence of firing rate on *SA* seen across CD morphologic space (compare Figure 8A and 8C). Instead, firing rate depended more on the axial resistance (*AX*) between the soma and the dendrite (*AX* ∝ *L*/*D* ^2^; see Materials and Methods; Figure 8C black line). Coupling between the soma and dendrite was weak when *AX* was high (upper left; high *L*, low *D*) and strong when *AX* was low (right, large *D*) [11,21]. Accordingly, models fired quickly in the upper left of the space, minimally affected by the inhibition-dominated dendrite, and more slowly to the right of the space, where reduced dendritic *SA* caused an increase in (mostly inhibitory) dendritic CDs. A total of 102 candidate models were identified across this space (colored symbols, Figure 8D).

#### Firing rate sensitivity across CN morphologic space.

Figure 8D shows sensitivities of spontaneous firing rate to active parameters *R_Ca_* and 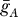 (top row) and to morphologic parameters *D* + *SA* / *CN* and *L* + *D* / *CN* (bottom row) for candidate models in CN morphologic space. Compare these to Figure 8B across CD space. Sensitivities to active parameters were similar in sign and relative magnitude to those across the CD morphologic and conductance spaces. Although sensitivities to morphologic parameters were smaller than in CD space, sensitivities to *D* + *SA* / *CN* and *L* + *D* / *CN* were larger than those to almost all active parameters for about half the candidate models with smallest *SA* (filled circles, Figure 8D, bottom row). In these models, only sensitivities to 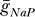 and 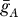 were larger. Sensitivities increased as firing rates decreased across space, but this trend did not follow decreasing *AX* as much as it did increasing *SA* (compare Figure 8A and 8C with arrows in Figure 8D). Thus, the principal sensitivity directions of the CD and CN morphologic spaces were similar (compare firing rate sensitivity to *R_Ca_*; arrows, Figure 8B and 8D).

#### Firing rate gain across CD morphologic space.

**Figure 9:**
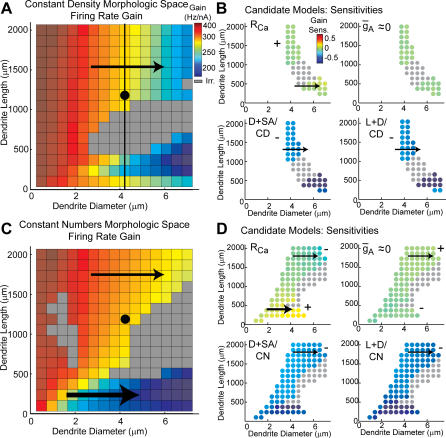
Firing Rate Gain Sensitivity Across Constant Density and Constant Numbers Morphologic Spaces (A) Colormap representing baseline firing rate gain across CD morphologic space (colorscale 0–400 Hz/nA; top right); the gain of models firing irregularly at 300 or 600 pA was not calculated (gray cells). Black vertical line denotes models with the default value of *D*; arrow indicates that *D* was the main driver of baseline gain. (B) Gain sensitivity to *R_Ca_* and 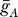 (top row) and to *D* + *SA* / *CD* and *L* + *D* / *CD* (bottom row). Models shown in gray fired irregularly. Arrows indicate the principal sensitivity direction with increasing *D*. Sensitivity to 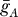 was near zero throughout the space. Analogous colormaps across CN morphologic space are shown for (C) baseline firing rate gain and (D) gain sensitivity to parameter perturbations among candidate models. Arrows in (C) show that decreases in baseline gain followed *D*; the principal sensitivity directions in (D) follow this trend. Across constant number space, sensitivities to active parameters often had opposite sign for large versus small *L* values (“−” versus “+” in [D]). Among the candidate models, gain was more sensitive to *D* + *SA* than to all active parameters except 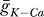 and sometimes 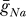 .

Most models across CD morphologic space fired regularly. A connected subset of these models fired doublets in response to 300–600 pA current injections (gray cells, Figure 9A). As with optimized models, such models were included in firing rate sensitivity analysis but were omitted for gain. Inspection of the relative timing of somatic and dendritic spiking revealed that the bursting mechanism lay within the soma of these models, and that dendritic membrane potential simply followed the somatic time course (unpublished data). While spontaneous firing rate largely followed *SA*, baseline gain exhibited a strong dependence on *D* (black vertical line, arrow) and minimal dependence on *L*, indicated by the vertical stripes of iso-gain values parallel to the *L* axis.

#### Firing rate gain sensitivity across CD morphologic space.

As across conductance space, gain was most sensitive to the inhibitory parameter 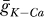 and excitatory parameters 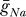 and 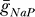 across CD morphologic space. Gain sensitivity was positive to excitatory and mixed parameters and negative to inhibitory ones. Figure 9B compares gain sensitivities to active parameters *R_Ca_* and 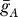 (top row) and morphologic parameters *D* + *SA* and *L* + *D* (bottom row) for candidate models across the space. The principal sensitivity direction to active parameters followed an increase in *D*, as shown by the arrow in Figure 9B, top left.

Among the candidate models, gain was more sensitive to the morphologic parameter *D* + *SA* than to all active parameters except 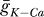 and sometimes 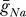 . For models with small *L*, sensitivities to *L* + *D* and *L* + *SA* were also high. Sensitivity to *L* + *SA* was positive for a few models; otherwise, sensitivities to all morphologic parameters were negative everywhere (Figure 9B, bottom row). The principal gain sensitivity direction to morphologic parameters, increasing as *D* increased, was the same as to active ones (compare arrows, Figure 9B, top and bottom rows).

#### Firing rate gain across CN morphologic space.

Two disjoint regions contained models that fired irregularly upon depolarization across CN morphologic space (Figure 9D, gray cells), and were omitted from the gain analysis. As seen in CD space, bursting occurred first at the soma then propagated to the dendrite for the region with large *D*, where somatic and dendritic membrane potentials were tightly coupled. In the second bursting region where *D* was small (and axial resistance high), somato-dendritic coupling was weak. In these models, bursting was caused by a delayed dendritic calcium spike that quickly repolarized the soma after its initial AP [21] (not shown). In the remaining models, gain decreased with *D* in two populations separated by their baseline gain values (Figure 9C, thick versus thin arrows). The low gain population included most models with small *L*, reflecting an increase in dendritic channel densities relative to the original morphology (black dot, Figure 9C) that was dominated by inhibitory parameters 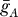 and 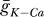 .

#### Firing rate gain sensitivity across CN morphologic space.

Figure 9D shows the sensitivity of firing rate gain to active parameters *R_Ca_* and 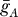 (top row) and to morphologic parameters *D* + *SA* and *L* + *D* (bottom row) for candidate models in this space. Similar to CD space, gain was more sensitive to all morphologic parameters than to all active parameters except 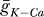 and sometimes 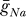 *.* The principal sensitivity direction to all parameters following increased *D* (compare Figure 9D with 9B). In addition to this trend, sensitivity to active parameters reversed sign as *L* decreased (arrows, Figure 9D, top left).

## Discussion

We have designed a novel method to quantify the sensitivity of neuronal output to different classes of model parameters. We applied the method to a model of neurons from goldfish Area II, which exhibit and likely contribute to persistent activity during eye velocity storage, a simple model of working memory. We examined how sensitivities to active and morphologic parameters varied among similarly firing models across the parameter space. Our results identified parameters contributing to functional homeostasis: some that were restricted to limited domains, and others that act globally. The sensitivities can be used to predict which parameters to perturb, and by how much, to compensate for a change in other parameters in order to maintain functional homeostasis. Conceptually simple but computationally rigorous, our method can be used to characterize the sensitivity landscape defined by any biological model, and to predict compensatory mechanisms over widely varying regions of parameter space.

### Sensitivities Compare Influences of Different Classes of Parameters

The sensitivity landscape of a model output variable (here, neuronal firing rate and gain) to each parameter was defined by the normalized sensitivity coefficient function (Equation 7, Materials and Methods), evaluated across parameter space. Sensitivity landscapes indicate domains where an increase in a parameter will either increase or decrease model output (where its sensitivity is positive or negative), and where the parameter's impact is high or low (where the magnitude of its sensitivity is large or small). Interactions between two or more parameters are likely to occur in domains where sensitivities to all those parameters are high. Thus, comparing the sensitivity landscapes to different parameters can elucidate which parameters might interact to control a certain behavior. Such information is essential for understanding mechanisms underlying homeostasis, widely observed in neuroscience [2–6,20,44] and in other biological systems [45–47].

Significantly, we found that while similar dynamics were generated from different parameter sets across the space, the *influence* of each parameter on these dynamics varied. This was shown here for a simple neuronal compartment model, but our methods can be used to study any computational model. Sensitivities of firing rate and gain to the active parameters 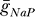 and 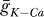 were high throughout the space; these results are supported by experiments [5,43,48], including in the vestibular nucleus, which is related functionally to Area II. Sensitivities to some parameters (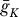 , 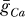 ; *L* + *SA*) were universally low, while sensitivities to all other parameters (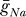 ,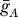 , *K_p_*, *R_Ca_*, *L* + *D*, *D* + *SA*), even morphologic ones, were high across certain domains of space. The similarity of these trends over morphologic parameter space confirmed the robustness of the sensitivities to wide variation in dendritic morphology.

**Figure 10:**
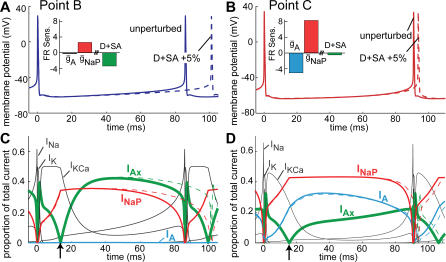
The Relative Size of Intrinsic Currents Determined the Size of Parameter Sensitivities (A) The unperturbed somatic voltage trace of the model labeled “B” in Figures 3A and 4D (solid line), compared with the voltage trace after a 5% increase in *D* + *SA* (dashed line). Inset barplot shows that spontaneous firing rate sensitivity of this model to 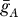 was low (blue), but high to 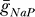 (red) and *D* + *SA* (green). (B) Somatic voltage traces of the model labeled “C” in Figures 3A and 4D. Labeling scheme is analogous to that in (A): the unperturbed model (solid) is compared with a 5% *D* + *SA* increase (dashed). Here, firing rate sensitivity was high to 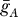 and 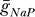 but low to *D* + *SA.* (C–D) The absolute value of each intrinsic current, normalized by the absolute sum of somatic membrane and axial currents at each time step, for the models shown in (A) and (B), respectively. The Na, K, and K–Ca currents are shown in gray (*I_Na_*, *I_K_*, *I_K-Ca_*). Also shown are A-current and NaP current (*I_A_*, determined by 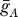 , in solid blue; *I_NaP_* determined by 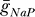 in solid red) and the somato-dendritic axial current (*I_Ax_*, determined by morphologic parameters, in heavy solid green). Black arrows indicate artifactual inflection points creased by taking the absolute value, when the unnormalized current reversed sign. For both points B and C, increasing *D* + *SA* delayed the next AP and reduced firing rate by increasing *I_Ax_*; *I_A_* and *I_NaP_* were also perturbed through their interactions with *I_Ax_* (dashed colored lines). (C) At point B, *I_Ax_* dominated during the ISI, so that the *D* + *SA* perturbation had a large effect on *I_Ax_*, and *I_NaP_*. As a result firing rate sensitivity to *D* + *SA* was high. (D) At point C, *I_Ax_* was relatively small throughout most of the ISI. Hence, increasing *D* + *SA* had a small effect on the intrinsic currents overall, so that firing rate sensitivity to *D* + *SA* was low.

Sensitivities of firing rate to all parameters varied most along the same principal directions: with 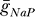 , 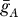 , *R_Ca_*, and 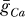 , and with dendritic *SA*. For firing rate gain, the principal sensitivity directions were relatively similar. Intrinsic currents controlling the neuron's membrane potential, and therefore its proximity to AP threshold, increased most along these directions, as illustrated below (see The Combination of Intrinsic Currents Determines Neuronal Firing Sensitivities, and Figure 10). As the membrane potential remained closer to AP threshold along this direction, perturbation of any parameter could more easily affect firing rate, so that sensitivity of firing rate and gain to all parameters increased along this direction. Thus, principal sensitivity directions indicate those “principal parameters” that contributed highly to the measured output over the space as a whole. Such principal parameters represent groups of parameters that must be closely tuned to maintain a certain level of output, and those that can compensate globally for changes in other parameters.

For illustration, we restricted this study to sensitivities of active and simple morphologic parameters, and to two of the many measures of neuronal function. Evidence suggests that sensitivities to other parameters, such as those describing passive cable properties, ion channel kinetics, and dendritic channel densities, may also be high [22,49]. Other firing rate and gain sensitivity trends may also exist along dimensions of parameter space that we did not explore. Importantly, sensitivity depends on the output being measured, like bursting, spike frequency adaptation and synaptic integration. The most appropriate output measures for a particular study will depend on the type and function of the neuron being analyzed. In general, sensitivities of somatocentric functions such as firing rate are likely to be highest to channel kinetics [49] and dendritic channel density gradients and intercepts (Figure S6), while sensitivities of dendritic functions such as AP backpropagation and propagation and integration of synaptic inputs are likely to be highest to passive parameters [22] and dendritic channel density gradients. Morphologic parameters are likely to influence both somatocentric and dendritic functions [11,13,22].

Maintenance of stable firing behaviors is important throughout the brain. The firing rates of Area II and central vestibular neurons, for example, encode precisely tuned eye velocity in response to a given angular head velocity. The fidelity of this match between oculomotor response and head velocity stimulus is crucial for maintaining accurate gaze control during locomotion. Our analysis predicts that individual Area II neurons could maintain a target firing rate stably over time by simultaneously regulating persistent sodium (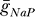 , high positive sensitivity) and K–Ca (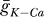 , high negative sensitivity). This prediction is supported by recent experiments in Purkinje cells and the stomatogastric ganglion [5,50]. Equivalently, a neuron could maintain a target input–output response by oppositely regulating sodium conductance densities (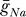 ) and dendritic diameters (*D* + *SA*), combining morphologic and active properties in appropriate proportions. The wide range of morphologic features observed among Area II neurons with similar firing properties [30] is consistent with this prediction.

### The Combination of Intrinsic Currents Determines Neuronal Firing Sensitivities

Neuronal firing rate reflects how frequently membrane potential in the spike initiation zone (here, the soma) crosses AP threshold. This frequency is strongly affected by currents that are active during the ISI, after the refractory period following an AP. We focus on two kinds of intrinsic currents: “membrane currents” flowing across the membrane, driven by active parameters, and “axial currents” between the soma and dendrite, which are most influenced by morphologic parameters. Parameter perturbations alter firing rate and gain by influencing these intrinsic currents, which constantly interact to determine membrane potential. In Figure 10, comparison of the relative sizes of these intrinsic currents provides a mechanistic explanation of the observed sensitivity trends. Figure 10A and 10B shows one ISI of the models marked (B,C) in Figure 4. Figure 10C and 10D illustrates the relative size of several intrinsic currents that are active during the ISIs at points B and C, respectively. The Na, K, and K–Ca currents are in gray (*I_Na_*, *I_K_*, and *I_K-Ca_*), NaP is in red (*I_NaP_*), A-current is in blue (*I_A_*), and somato-dendritic axial current (*I_Ax_*) is in green. We show the absolute value of each normalized current. This simplifies comparison of the different currents, but causes artifactual inflection points as currents change sign (e.g., black arrows in Figure 10C and 10D).

At point B where the parameter 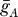 was low (Figure 4D; Equation 2), *I_A_* was negligible, while both *I_NaP_* and *I_Ax_* were large during the ISI. Note that *I_Ax_*, the only current directly affected by morphologic perturbations, was the largest current over most of the ISI. The dashed lines in Figure 10 show the effect on model output of a 5% increase in *D* + *SA*, which decreased axial resistance by 9.3% (Equation 8). The increased *I_Ax_* hyperpolarized the soma, perturbing membrane currents as they interacted with *I_Ax_*, and reduced firing rate (Figure 10A and 10C). Because of the prominence of *I_Ax_*, then, firing rate sensitivity was higher to *D* + *SA* than to any active parameter (Figure 10A, inset). This was true for most of the optimized models (filled circles, Figure 4D). In contrast, at point C, where 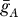 and 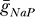 were large (Figure 4D), membrane currents *I_NaP_* and *I_A_* were the largest currents over most of the ISI (Figure 10D), dominating *I_Ax_*. Morphologic perturbations therefore had little influence on the ISI (Figure 10B and 10D, dashed lines). For this and three other optimized models (open squares, Figure 4D), firing rate sensitivity was higher to most active parameters than to morphologic ones (Figure 10B, inset).

This intuitive understanding of firing rate sensitivities based on the combination of intrinsic currents explains the sensitivity trends that we observed. For example, sensitivity to morphologic parameters sometimes changed sign across parameter space (e.g., Figure 4D). This occurred because perturbations to morphologic parameters with, say, an inhibitory effect on *I_Ax_* had an excitatory effect on some membrane currents, and the size of those membrane currents changed across parameter space. Also, sensitivity to morphologic perturbations with constant channel densities or numbers had opposite sign when dendritic currents were predominantly inhibitory (Figure S6A), but not when excitatory and inhibitory currents were balanced (Figure S6B). Finally, as noted above, the principal firing rate sensitivity directions indicated the direction in which the membrane currents dominating the ISI (membrane currents *I_A_*, *I_NaP_*, and *I_K-Ca_*) increased most. These currents were most increased by variations in 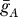 , 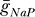 , 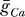 , and *R_Ca_* across conductance space, and in *SA* across morphologic space.

Principal sensitivity directions for gain were similar to those for firing rates, but not identical. External current injection altered the activation of voltage-dependent intrinsic currents, affecting the combination of currents during the ISI. Because gain reflected firing rate over several levels of injected currents, its principal sensitivity directions gave the average direction in which intrinsic currents increased over the different injection levels. Although principal sensitivity directions of other output variables may be oriented differently from firing rate or gain, we expect their principal directions to reveal how the mechanisms driving those outputs change most.

### Building upon Previous Approaches

Sensitivity analysis has previously been used to quantify the influence of each parameter on model output in one of two ways [33]: locally, at a single point in space; or globally, summarizing sensitivity across the entire space. A synthesis of these approaches, computing local sensitivity coefficients globally across the space, allowed us to investigate the shape of the sensitivity landscape. The wide variation in sensitivity landscapes suggested that global sensitivity measures across the parameter space are likely insufficient to characterize the domain-specific interactions that we observed. The global trends and interactions identified by our first-order sensitivity analyses provide a foundation on which higher-order analyses [33,35] can be built.

Computational studies searching over conductance space have discovered multiple parameter sets that represent plausible neuronal models, highlighting the existence of interactions within model neurons and in model circuits [2,3,6,20,51,52]. Goldman et al. identified sensitive directions of space along which firing changed from silent, to spiking, to bursting [20]. Our principal firing rate sensitivity directions were consistent with this finding, and also extended to firing rate gain. The silence, regular spiking, and bursting we observed across connected regions of parameter space have also been reported by others [2,20,52]. In our model, bursting was due to the buildup of Ca^2+^ and the activation of *I_K-Ca_*, either intrinsically at the soma [2,20,53] or in a weakly coupled dendrite that influenced somatic firing [21]. We expect that both kinds of bursting exist over all three parameter spaces we explored, for appropriate ranges of parameters. We did not compute sensitivities when a model was either silent or bursting at a given current level, to avoid the artifactually high sensitivities that can arise at the borders of such regions. If a model fired bursts upon depolarization but spontaneously fired single action potentials, we assumed its spontaneous behavior provided insight into the mechanisms that underlie spontaneous firing. Such models were included in the analyses of sensitivity to firing rate but not gain.

As in several prior studies [11,21,22], we interpreted morphologic features as model parameters, allowing us to explore how morphology influences neuronal function. The novel insights of our work arise from our simultaneous exploration of sensitivity landscapes defined by morphologic and active parameters. The results from our simple model suggest that morphology can influence firing as much as active parameters do, through its contributions to axial and dendritic currents that impact the soma. For example, when intracellular Ca^2+^ removal rates were low (*R_Ca_* < 0.33 ms^−1^), Ca^2+^ built up in the dendrite. In such models, perturbations of dendritic morphology strongly influenced this Ca^2+^ buildup, sensitively affecting firing rate and gain. However, these high sensitivities are certainly model dependent, decreasing when membrane conductance is high and losing dependence on *R_Ca_* when 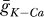 is low.

### Potential Limitations of the Method

While our method provides a systematic way to evaluate how different parameters contribute to model output, it is subject to certain limitations when applied to complicated models. For example, our method minimizes computational burden by evaluating only first-order sensitivities across the parameter space. Such sensitivities may lose their predictive value outside a local neighborhood of each point, and cannot probe interactions among parameters directly. Higher-order derivatives or variance-based methods [33,35] may better analyze the behavior of complex models, but at increased computational cost.

**Figure 11:**
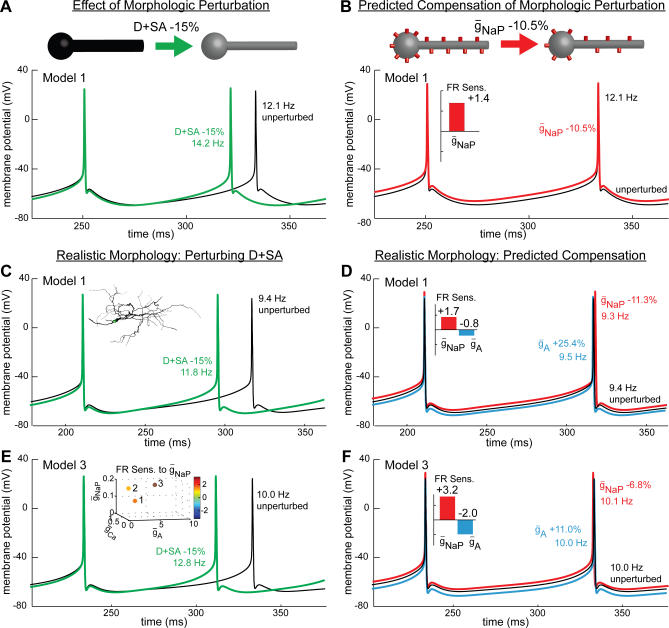
Using Sensitivities To Predict Parameter Compensations for Firing Rate Homeostasis in the Reduced and Realistic Morphologies (A–B) Parameter perturbations of Model 1 from subspace E (Figure S5, and inset of [E] below). (A) A 15% reduction of *D* + *SA* increased firing rate to 14.2 Hz (thick green line) relative to the unperturbed model (12.1 Hz; thin black line); a 14.8% reduction is needed to return to the unperturbed firing rate. (B) As predicted from its firing rate sensitivity (inset barplot), a 10.5% decrease in 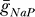 compensated almost exactly for the effect of the *D* + *SA* perturbation on firing rate in (A) (see Discussion for details). The red trace, shifted up slightly along the vertical axis for visualization, overlays the unperturbed voltage trace very closely. (C–F) Homeostatic parameter compensations can also be predicted in models with realistic morphology. (C) When Model 1 from subspace E was simulated in the realistic AII morphology, a 15% reduction of *D* + *SA* increased firing rate by 20.3% relative to the unperturbed value. (D) The compensatory perturbations for Model 1 of two different active parameters (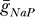 in red, shifted vertically up; 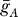 in blue, shifted down) were predicted from their firing rate sensitivities (inset barplots). The blue and red compensated traces overlie the unperturbed (black) trace very closely, demonstrating excellent compensation. (E–F) Compensatory perturbations predicted from sensitivities computed at a different point in parameter space: Model 3 of subspace E, implemented in the realistic morphology with an analogous 15% reduction of *D* + *SA.* Inset in E shows the location of Models 1, 2, and 3 in the [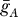 , 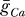 , 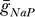 ] subspace (also see Figure S5A). For Model 3, firing rate sensitivities to 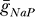 and 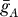 were about twice as large as for Model 1. Accordingly, the predicted magnitudes for Model 3 were about half of those for Model 1; compare (D) and (F). For both models, the close match of red (shifted up), blue (shifted down), and black traces demonstrate that the predicted perturbations of *either* active parameter compensated almost exactly for the effect of the *D* + *SA* reduction ([C] and [E]), even for large predicted increases of 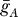 . See Discussion for details.

In the present study, our morphologic model comprised a soma and cylindrical dendrite. We chose a simply parameterized morphology to illustrate a comparison between sensitivities to morphologic and active parameters. While it clarified essential morphologic mechanisms that influence somatic firing patterns, this simple model cannot reproduce all the subtle interactions that shape neuronal function in realistic morphologies. Fortunately, the model reduction is not a necessary step in our analysis. As demonstrated below (Figure 11), our method can be applied to models with more realistic morphologies. The trends identified here may change when analyzing sensitivity to more complex morphologic features of dendrites, such as tapering, branching, and varied dendritic lengths. Nonetheless, supported by findings that dendritic current flow can significantly affect gain control and excitability [54–56], our results indicate that morphology can play a key role in functional homeostasis.

As with any analysis technique, using our method to explore a computational model becomes more challenging as model complexity increases. Computational demand scales with both the number of parameters and the number of outputs being analyzed. Moreover, it may be difficult to parameterize some intrinsic properties of neurons, including local morphologic features of dendrites or spines, or spatially extended membrane properties, to allow their systematic variation. We will examine such parameters, and efficient methods to analyze them, in future studies.

### Predicting Homeostatic Parameter Compensations in Real Neurons

Experimental and computational studies alike have shown that cellular mechanisms contribute to functional homeostasis (see Marder and Goaillard [7] for review), but how can we identify which of these mechanisms, and in what proportions, actually do trade off against each other in a given neuron? As in past studies [2,3,6,20,51], we have identified some global tradeoff mechanisms through trends in the parameter space locations of similarly behaving models, including a correlation between 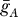 and 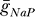 supported by a recent study [50]. Identification of homeostatic mechanisms is an active field of research, with promising techniques beginning to emerge [6,52].

In this vein, sensitivity landscapes provide new model information that can predict global and domain-specific tradeoffs. Parameters with high sensitivity but opposite sign over substantial regions of parameter space (e.g., 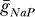 and 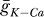 ) could represent global tradeoff mechanisms, similarly with parameters that define principal sensitivity directions. At the same time, the sensitivity landscapes also seem to *exclude* 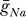 , 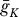 , and 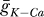 from purely domain-specific interactions, since we found that sensitivities did not change significantly across these dimensions of parameter space.

In individual models, the sensitivities allow quantitative predictions of how large a change in one parameter is needed to counteract a given change in another. These compensations could be made between two parameters, or among groups of parameters, and could include parameters with entirely different dimensions, like morphology and active membrane conductances. Such compensations may be used during development in some neuronal systems, in which function is maintained despite morphologic changes [4,39]. Figure 11A and 11B uses sensitivities to predict how an active parameter can compensate for a morphologic change. The “target” firing of an unperturbed model from subspace E (Model 1 in Figures S5A and 11E inset) is shown in black; the effect of a 15% decrease in *D* + *SA* to this model is shown in green. To return to its target level, the firing rate of the reduced *D* + *SA* model must decrease by 14.8% (from 14.2 Hz to 12.1 Hz). From a small perturbation to 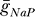 in the reduced *D* + *SA* model (Figure 3B and Equation 7), we found that sensitivity of firing rate to 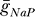 was +1.4 (Figure 11B inset). Using the equation for the normalized sensitivity coefficient (Equation 7), we predicted that the target firing rate can be restored by a −14.8% / +1.4 = −10.5% change in 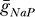 . This compensatory perturbation is shown in red in Figure 11B, overlaid with the unperturbed model in black (shifted slightly to aid visualization): the predicted 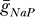 perturbation compensated almost exactly for the reduction in *D* + *SA*.

Such compensatory predictions can also be made in models with realistic neuronal morphologies. We applied the parameter values from Model 1 of subspace E to the 3-D Area II neuron morphology from Figure 1A. In this model, a 15% reduction in *D* + *SA* throughout the dendritic tree increased firing rate from 9.4 Hz to 11.8 Hz (Figure 11C, black; unperturbed versus green traces). To return to the target level, the firing rate of the *D* + *SA*–reduced model must decrease by 20.3%. Figure 11D shows how perturbing either of two different active parameters could achieve this decrease. From small perturbations, the firing rate sensitivity of the *D* + *SA*–reduced model to 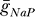 was +1.8, and sensitivity to 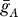 was −0.8 (Figure 11D inset). Again using Equation 7, these sensitivities predicted that either a −20.3% / +1.8 = −11.3% change in 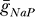 or a −20.3% / −0.8 = +25.4% change in 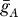 should compensate for the *D* + *SA* reduction. Figure 11D shows that either of these perturbations compensated almost exactly for the *D* + *SA* reduction (colored versus black traces), even though such large perturbations were predicted. The accuracy of these two different compensations suggests that a reasonable compensatory prediction can be made for *any* parameter with high sensitivity, or even with combinations of such parameters.

Sensitivities and compensations of realistic models elsewhere in the parameter space further demonstrate the general utility of our method. For a second model (Model 2 in Figure S5A and Figure 11E inset), the firing rate sensitivities to 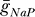 and 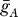 were similar to those of Model 1; note the colorscale in the inset of Figure 11E. Accordingly, the predicted compensations of either 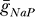 or 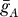 to counteract the *D* + *SA* reduction in Model 2 were similar in sign and magnitude to those for Model 1 (Figure 11D; Model 2 results not shown). For a third realistic model (Model 3 in Figures S5A and 11E), a 15% reduction in *D* + *SA* increased firing rate from 10.0 to 12.8 Hz (Figure 11E, green versus black traces). A 21.9% decrease in firing rate of the *D* + *SA*–reduced Model 3 would restore the target firing level, similar to the decrease needed for Model 1. However, firing rate sensitivities of Model 3 to 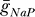 and 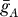 were about twice what they were for Model 1 (Figure 11E, colorscale in inset; compare inset sensitivity bar graphs, Figure 11D and 11F), increasing along the same principal sensitivity direction as the simple morphologic model. Thus, the predicted perturbations of either 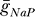 or 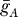 to compensate for the Model 3 *D* + *SA* reduction were about half the size of those for Model 1 (Figure 11D and 11F). These examples show that the magnitudes of compensatory predictions correspond directly to trends identified in the sensitivity landscapes, and that the findings from our cylindrical dendrite model are relevant to more complex morphologies.

Sensitivity analyses are often performed on kinetic parameters or on concentration levels of a substance; to our knowledge, this is the first time sensitivities to morphologic parameters have been evaluated. Our method enables us to trade off morphology quantitatively against intrinsic parameters of completely different dimensions, as shown in Figure 11. Such quantitative predictions can elucidate potential mechanisms of functional homeostasis used to maintain target activity throughout the brain, despite morphologic changes that occur with development [4] and learning [57]. This may even suggest intrinsic mechanisms to counteract morphologic changes underlying cognitive decline in aging and neurodegenerative diseases [40]. In short, the quantitative prediction of parameter compensations produced by our method will be useful in many areas of computational biology and biomedicine.

## Materials and Methods

Throughout the text, firing rates are reported as mean ± standard deviation.

### Morphologic data.

A neuron from Area II of the goldfish hindbrain was electrophysiologically characterized in vivo and injected with biocytin to visualize its morphology (Figure 1A). Histological processing proceeded as described in Straka et al. [30]. The neuron was traced in 3-D using Neurolucida (MicroBrightField), then converted into .swc format [58]. The morphologic dimensions were corrected to account for tissue shrinkage and compression, estimated at 10% in the *X* and *Y* dimensions, and 47% in the *Z* dimension from the difference in tissue thickness before and after histology, mounting, and coverslipping. The data were examined in 3-D using interactive custom software (NeuroGL; NeuronStudio [59,60]) for broken dendrites and *Z*-axis artifacts that can occur with manual tracing. Artifacts were corrected manually before length and surface area measurements were made.

### Mathematical model.

The NEURON simulation environment [61] was used for this study. The compartment model included a soma (length and diameter 29.2 μm, one compartment) and cylindrical dendrite (length 1183.6 μm, diameter 4.38 μm, 13 compartments) whose dimensions conserved the shrinkage-corrected maximum length and surface area of the traced Area II neuron. It was assumed that the somatic compartment included the spike initiation zone. A simulation time step of 0.05 ms was used. Based on a recent single compartment model of vestibular nucleus neurons [41,42], a passive leak (Lk) and six active conductances were included in the soma. The transient sodium (Na) and delayed rectifier potassium (K) currents were described by the Fitzhugh-Nagumo model [62,63]. The remaining active currents (transient A-type potassium [A], high-voltage activated Ca^2+^ [Ca], K–Ca, and persistent sodium [NaP]), were described by Hodgkin-Huxley formalism. With these currents, specific membrane capacitance *C_m_* (μF/cm^2^), dendrite diameter *D* (cm), axial resistivity *R_a_* (Ω · cm) and injected current (*I_inj_*), membrane potential *V* was given by

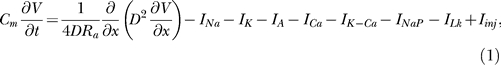
where the current *I_s_* for each ion species *s* was described by

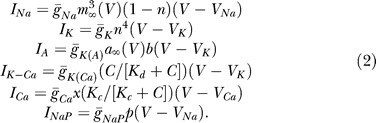



Here, 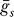 was the maximal conductance of a membrane patch (mS/cm^2^), with reversal potentials *V_s_* (mV) and constants *K_d_* = 0.0005 mM and *K_c_* = 1 mM as in Av-Ron and Vidal [41]. Kinetics of the channel activation and inactivation variables {*a*, *b*, *m*, *n*, *p*, *x*} are given by Equations 4–6 below (Ion channel kinetics). For the passive leak current *I_Lk_*, 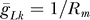 where *R_m_* was the specific membrane resistance (kΩ·cm^2^). Passive parameters *C_m_* = 1.0 μF/cm^2^, *R_a_* = 150 Ω·cm, and 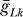 = 0.3 mS/cm^2^ (*R_m_* = 33.33 kΩ·cm^2^) were used in all simulations [11,13,64]. The dynamics of intracellular Ca^2+^ concentration, *C* (mM)*,* were described phenomenologically by the first-order equation

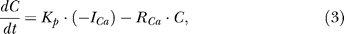
where the influx parameter *K_p_* (M · cm^2^/mC) converted Ca^2+^ current to concentration, and *R_Ca_* (ms^−1^) represented removal of Ca^2+^ from the cytoplasm.

### Ion channel kinetics.

Equations for channel kinetics were identical in all compartments of the model, with parameter values equal to those of Av-Ron and Vidal's Type B model [41]. All activation and inactivation variables were described by a representative equation:

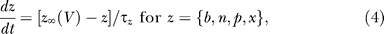
where *τ_z_* represented the time constant, and 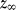 the steady-state function, defined by

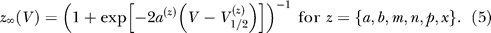



In each case, 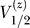 was the half-activation voltage, and *a* ^(*z*)^ controlled the slope of the sigmoid at this midpoint. All time constants *τ_z_* were constant parameters, except for *τ_n_*:

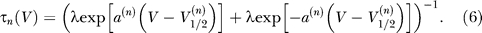



The parameter *λ* relates to the temperature of the preparation, which alters the peak value of *τ_n_*.

### Ion channel distributions in the active dendrite.

Since ion channel distributions in the dendrites of Area II neurons are unknown, we considered two cases: a passive dendrite (e.g., 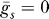 for all ion species except *Lk*; Figure 1B, left), and a dendrite supporting active backpropagation, as observed in other neurons [65,66] (Figure 1B, right). Each channel distribution in the active dendrite was defined by a slope, or gradient, and an intercept that scaled the dendritic conductance density relative to its somatic value linearly with distance. The slopes and intercepts of dendritic conductance densities were chosen manually to approximate the reduced amplitudes and increased widths of backpropagating APs. Dendritic channel distributions either remained constant (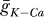 and 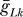 ), decreased to zero (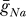 , 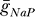 , 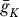 , and 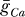 ), or increased with distance from the soma (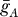 ), consistent with observations in several brain areas [14,18,19]. Slopes and intercepts of conductance densities are shown in the top of Figure 1E. Backpropagation of somatic APs into the dendrite, as a result of these dendritic distributions, is shown at the bottom of Figure 1E. Compare profiles of the somatic AP (red trace) with the AP as it backpropagates through the dendrite (black traces).

### Automated parameter optimization.

Extracellular recordings have reported spontaneous firing rates in Area II neurons of 10.4 ± 5.8 Hz in vivo [31]. In vitro intracellular recordings have identified neurons firing spontaneously at up to 70 Hz, some with a biphasic AHP characteristic of Type B vestibular neurons (G. Gamkrelidze, personal communication). Starting from a single compartment model of Type B vestibular neurons [41], we used a systematic search to identify parameters of the maximal conductances of the ion channels and calcium dynamics producing models best exhibiting these properties, particularly the biphasic AHP shape. The parameters [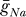 , 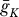 , 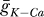 , 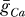 , 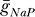 , *K_p_*, *R_Ca_*] were varied over [30%, 60%, 100%, 130%, 160%, 200%] of the values used by Av-Ron and Vidal [41], holding 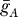 = 0 [41]. Of the nearly 280,000 models sampled by this systematic search, one was selected which best reproduced firing rates and regularity of Area II in vivo [31] (Table S1). This single compartment model, firing spontaneously at 8 Hz, comprised the synthetic “target” data against which morphologic models were matched [42].

We quantified the fits of each model to target data with a linear combination of two kinds of errors: time-aligned AP shape error *E_AP_* [42] (Figure S8A), and time-varying firing rate (Figure S8B). For *E_AP_*, we calculated the root mean squared difference between voltage traces for each target and the corresponding model AP, after aligning their peaks in time; the average of these differences comprised *E_AP_*. To calculate the firing rate error, we computed the instantaneous firing rate, defined as the reciprocal of each ISI, between successive APs of each voltage trace. Time-varying firing rate was measured by fitting a straight line to the instantaneous firing rates versus time. The intercept represented the neuron's baseline firing rate, and the slope captured a first-order time dependence to fit the spike frequency adaptation observed in Area II neurons (G. Gamkrelidze, personal communication). The time-varying firing rate error included one term for the slope error (*E_FR(Slope)_*), and another for the overall error (*E_FR(Area)_*). A full description of the fitness function, including these and additional error terms and their associated weights, is provided in Protocol S1. The value of the fitness function was calculated for each of two current magnitudes (0 pA, 100 pA), injected from 200–700 ms of simulation time; total fitness was the sum of these errors. We found this fitness function superior to the phase-plane fitness function [6,67] for matching AHP shapes, which are distinctive features of Type B–like Area II firing patterns.

We used constrained simulated annealing with recentering for accurate boundary management [42,68] to optimize values of 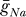 , 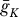 , 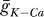 , 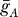 , 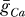 , 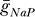 , *K_p_*, and *R_Ca_*, assuming a passive dendrite for simplicity. Simulated annealing searches are controlled by an initial annealing temperature *T_0_* and a cooling rate *r* that is applied every *K* iterations [69,70]. Maximal conductance and calcium dynamics parameters were varied within constraints described below and shown in Table 2. In 20 searches, five parameters were optimized, with [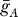 *,* 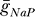 , 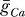 ] fixed at previous values [41,42], *T_0_* = 1,200, *r* = 0.9, and *K* = 1,000. In another 20 searches, seven parameters were optimized, with 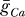 fixed at 0.001 mS/cm^2^, *T_0_* = 12,500, *r* = 0.9, and *K* = 1,400. In most searches, *K_p_* and *R_ca_* were tightly bound; relaxing this condition in 10 of the seven-parameter searches still identified matches qualitatively similar to the target data. Models were constrained to match the general AP shape, and approximate firing rates, of the synthetic target data. From the points identified at the end of the 40 independent parameter searches, the 15 optimized models with lowest error were selected for the sensitivity analysis (mean error, 14.9; Figures 3, 4, S2, and S3, and Dataset S1). Nine of these models were selected from the five-parameter searches each sampling about 164,000 points in parameter space; the best-fit models were identified after about 75,000 iterations. The remaining six optimized models were selected from the seven-parameter searches each sampling about 240,000 points, with best-fit models identified after about 105,000 iterations. A typical fit of an optimized model to target data is shown in Figure 2B. Although these models spanned a range of fitness values relative to the target data (Figures 2B and S8C), their firing rates over the fitted protocols were consistent with firing properties in Area II (spontaneous rates 8.9 ± 1.9 Hz).

### Parameter search constraints.

Boundary constraints on parameters optimized by the search included at least one order of magnitude around values used for the Av-Ron and Vidal Type B model [41]. These constraints are shown in Table 2, with references to other computational studies with parameters in this range. Since *K_p_* and *R_Ca_* varied over orders of magnitude in the literature (e.g., [6,20,41,71,72]), they were loosely constrained in the optimized and systematic searches.

To better constrain *K_p_* and *R_Ca_* in future studies, we returned to early studies from which these parameters were derived [71,72]. Equation 3 was derived from a model to describe the Ca^2+^ concentration in a thin shell near the membrane [71]. In the original model, *C* / (*A* · *d*) rather than *K_p_* multiplied the first term, where *d* is the shell depth, *A* is the patch surface area (in μm and μm^2^, respectively), and *C* a constant to convert current, time, and volume into Ca^2+^ concentration. Applying Faraday's constant and dimensionality arguments gives *C* = 0.0518, as derived in McCormick and Huguenard [72]. Because NEURON accounts for compartment surface area in its calculations [61], *K_p_* as used here depends on *d* but not *A*. Shell depths from 0.5 nm to 1 μm yield values of *K_p_* from 103.6 to 0.0518 M · cm^2^/mC; *K_p_* > 1 implies *d* < 51.8 nm.

The parameter *R_Ca_* is the inverse of a Ca^2+^ decay time constant [71]. Experimental time constants in several neuron types varied from 20 ms [73,74] up to 1 s [75], implying that plausible values of *R_Ca_* range from 0.001 = 1/1000 ms^−1^ to 0.05 = 1/20 ms^−1^.

### Perturbed parameters.

Perturbed parameters are summarized in Table 1. Morphologic perturbations *L* + *SA*, *D* + *SA*, and *L* + *D* were performed as described above with either densities or absolute numbers of dendritic ion channels held constant (Figure 1C and 1D). Soma size was not perturbed. When maintaining constant channel numbers, the active parameters *K_p_* and *R_Ca_* and passive parameter 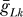 were unchanged. Holding morphology unchanged, we also perturbed “active” parameters, defined as maximal ionic conductances (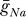 , 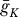 , 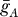 , 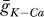 , 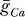 , and 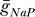 ), and parameters describing Ca^2+^ dynamics (*K_p_*, *R_Ca_*).

### Quantifying model output.

The firing properties of each model neuron were quantified by two output measures: spontaneous firing rate (Hz), and firing rate gain (Hz/nA). These output measures are representative of the type of data that can be analyzed with this method; other measures are possible. Spontaneous firing rate was defined as the reciprocal of the mean ISIs in the absence of external current injection. Firing rate gain was defined as the best-fit slope of firing rate versus injected current measured over a sequence of [0, 300, 600, 1000] pA steps of injected current (Figure 2A); these ranges were consistent with experiments in vestibular nucleus [76]. Because many active dendrite models responded nonlinearly to large currents, gains across conductance space were evaluated over injections of [0, 50, 100, 125, 150] pA. Each simulation first ran for 200 ms to allow for model initialization. Somatic current injections were applied at 200 ms. Output measures were estimated from APs occurring between 400–900 ms. Neuronal responses under somatic current injections were compared to those under dendritic synaptic input, for the 19 candidate models in subspaces A and E (Figure S5). A total of 45 excitatory synapses were evenly distributed throughout the dendrite, with synaptic conductances and kinetics taken from Rudolph and Destexhe [77]. Synaptic activations were modeled as independent Poisson processes, with mean frequencies of [1, 5, 10, 20, 30] Hz. Firing rates were computed over a simulation time of 5 s, and synaptically activated gain was defined as the best-fit slope of these firing rates versus synaptic frequency. The correlation coefficient of the somatic input-driven versus synaptic gains was computed for these 19 candidate models (*r* ^2^ = 0.992).

### Sensitivity analysis.

At each point *P* = {*p_i_*, *i* = 1,..,*n*} in *n*-dimensional parameter space, each parameter *p_i_* was perturbed by 1% while all others were held constant. The linearity of each output measure was confirmed for a representative set of model neurons; in many cases, linearity was still maintained under perturbations of 30% in each parameter. The sensitivity ∂*M*/∂*p_i_* of each model output measure *M* with respect to a parameter *p_i_* at the point *P* was estimated by finite difference. The normalized sensitivity coefficient [33],

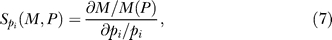
was approximated from these perturbations. The magnitude of 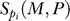 measures the percentage change in *M* given a 1% change in *p_i_*; its sign indicates whether this parameter perturbation has a positive or negative effect on the output measure *M*. Regression analyses over morphologic parameters showed that spontaneous firing rate was most sensitive to *D*, followed by *SA* (results not shown); therefore, sensitivities to morphologic perturbations were normalized by *D* for *D* + *SA* and *L* + *D* perturbations, and by *SA* for *L* + *SA*. Principal sensitivity directions, along which sensitivity to each parameter varied most, were identified visually.

### Systematic searches of parameter space.

Parameter values fixed in the systematic searches were representative of the locations of optimized models in parameter space. For the passive dendrite search, parameters 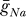 , 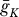 , and 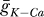 were fixed to 20, 10, and 2 mS/cm^2^, respectively. Upper and lower bounds for the search are shown in Table 3. The parameters (*K_p_*, *R_Ca_*, 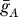 , 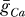 , and 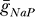 ) each assumed one of five values equally spaced between these constraints, yielding 5^5^ = 3,125 passive dendrite models across conductance space. Sensitivity analyses were performed on this entire dataset (Dataset S2).

Using these same parameters in the active dendrite search of conductance space yielded models with unrealistically deep AHPs. Therefore, the somatic values of 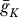 and 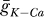 were changed slightly for the active dendrite search of conductance space, to 15 and 1 mS/cm^2^, respectively (Table 3). To explore more of this space, the number of grid points along 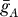 and the upper constraint on 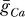 were doubled relative to the passive dendrite model search; grid sizes in other dimensions were unchanged. As a result, the active dendrite search of conductance space visited 8 × 9 × 5^3^ = 9,000 points (Dataset S3).

Area II–like candidate models were loosely defined as those firing spontaneously between 7–13 Hz, but with unrestricted gains. Some candidate models spontaneously fired single action potentials but burst upon depolarization; these were still classified as candidate models for spontaneous firing but were not used to analyze gain trends. The 7–13 Hz range was within the 4.6–16.2 Hz range observed experimentally in vivo [31] without deviating too far from the mean baseline firing rate. The active dendrite candidate model with lowest firing rate gain over the 0–1000 pA range (281 Hz/nA), although high, matched gains measured in vestibular nucleus neurons in vitro most closely (mean 102.7 Hz/nA [76]). In systematic searches of morphologic space, active parameters were fixed at the values from this model (Table 3; blue triangle marked ‘M' in Figure 6). Both *L* and *D* were varied over wide ranges, assuming 15 equally spaced values from zero to nearly twice their default values (Table 3). Such wide bounds for *D* assume that the cylindrical dendrite approximates an equivalent cylinder reduction of a neuron's entire dendritic tree [78]. These 225 models across morphologic space were visited twice, holding either dendritic channel densities or numbers constant (Dataset S4). The axial resistance *AX* between the soma and the first dendritic compartment (with length *L_0_*) was defined by

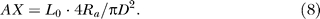



### Models for homeostatic parameter compensations.

Figure 11A and 11B was created using Candidate Model 1 in subspace E (Figure S5A). Applying its active and passive parameters to the 3-D morphologic data (Figure 1A), with dendritic channel densities as described above (Figure 1E), gave the model shown in Figure 11C and 11D. Models 2 and 3 in subspace E were also applied to the realistic morphology, with conductance parameters further tuned by hand to produce spontaneous firing (e.g., Figure 11E and 11F).

## Supporting Information

Dataset S1 Parameter Values of the 15 Optimized Models (16 KB GZ).

Dataset S2 Active Dendrite Models Visited During the Systematic Search of Conductance Space (3.3 MB GZ).

Dataset S3 Passive Dendrite Models Visited During the Systematic Search of Conductance Space (2.0 MB GZ).

Dataset S4 Active Dendrite Models Visited During the Systematic Search of Morphologic Space (381 KB GZ).

Figure S1Optimized Models Showed a Strong Correlation Between 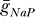 and 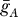

The values of 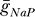 and 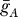 of the 15 optimized models were positively correlated across the entire [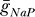 , 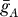 ] subspace.
(459 KB TIF)

Figure S2Sensitivity of Spontaneous Firing Rate to Parameter Perturbations for Optimized Models Across Conductance Space(A) Spontaneous firing rate of the optimized models as a function of their location in the [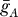 , 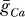 , 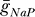 ] subspace. Each optimized model is represented as a circle whose color represents its spontaneous firing rate, according to the colorscale at right.
(B) Sensitivity of spontaneous firing rate to active parameters *R_Ca_* and 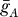 (top row, left and right) and morphologic parameters *D* + *SA* and *L* + *D* (bottom row, left and right). The principal sensitivity directions were similar for active and morphologic parameters across the space (compare black arrows in top and bottom rows).
(859 KB TIF)

Figure S3Negative Firing Rate Sensitivity to 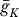 Was Induced by the K–Ca Current
(A) While 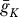 is intuitively considered an inhibitory parameter, in these models it had an excitatory effect. Compare unperturbed (black line) versus a 100% increase in 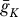 (red dashed line); firing rate sensitivity to 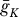 was positive (inset).
(B) Detail of the shaded region in (A). The unperturbed model is shown as black solid lines; the response to a doubling of 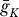 is shown as dashed red lines. Top, membrane potential: the increased 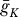 caused a deeper fast AHP. Middle, because of the hyperpolarized membrane potential, the increased 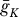 reduced Ca^2+^ influx into the neuron. Bottom, the reduced intracellular Ca^2+^ caused a smaller K–Ca current, so that the medium AHP in the perturbed model was smaller. With its membrane potential closer to AP threshold, the model with increased 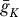 fired earlier than the unperturbed model. Induced perturbations of other intrinsic currents were not sufficient to explain the variation in firing rate (not shown).
(C) Flow chart of the mechanism underlying the positive sensitivity to 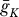 , demonstrated in (B).
(D) Validation of the mechanism: when the K–Ca current was removed from the model by setting 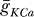 = 0, a 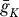 increase reduced firing rate. Compare black and red dashed lines. In models with little or no K–Ca current, firing rate sensitivity to 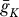 was negative, as expected intuitively (inset).
(1.0 MB TIF)

Figure S4Sensitivity of Firing Rate Gain to Parameter Perturbations for Optimized Models Across Conductance Space(A) Firing rate gain of the optimized models as a function of their location in the [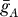 , 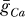 , 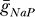 ] subspace (colorscale at right).
(B) Sensitivity of gain to active parameters *R_Ca_* and 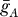 (top row, left and right) and morphologic parameters *D* + *SA* and *L* + *D* (bottom row, left and right). Filled circles show models for which sensitivity was greater to morphologic parameters than to six or more active parameters. Open squares show models for which sensitivity to morphologic parameters was less than to three or more active parameters. Arrows indicate similar principal sensitivity directions to 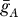 and *D* + *SA* across the space. There were no clear trends in the sensitivities to *R_Ca_* and *L* + *D*.
(828 KB TIF)

Figure S5Spontaneous Firing Rate Sensitivity Trends Depended Strongly on Calcium Dynamics Parameters(A) Sensitivity of spontaneous firing rate to *R_Ca_* across active dendrite conductance space (colorscale, top right). Shown are candidate models from subspaces E, F, A, and C (shown in [B], red dots). The parameter *K_p_* increases from the top row to the bottom (from subspace E to F; and from subspace A to C). Parameter *R_Ca_* increases from the left to right (from subspace E to A; and from subspace F to C). Arrows show the global sensitivity trend in each subspace: sensitivity magnitude increased with *R_Ca_* (thin versus thick arrows, from subspace E to A; and from F to C) but not *K_p_* (from subspace E to F; and from A to C). Filled circles are models where sensitivity to a morphologic parameter was greater than to all active parameters except 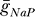 and sometimes 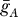 or 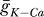 . Open squares are models where sensitivity to most active parameters was greater than to most morphologic parameters. Models labeled (1,2,3) are used to predict compensatory parameter perturbations in Figure 11.
(B) Candidate models identified during the systematic search of *passive dendrite* conductance space. Values of 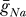 , 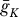 , 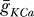 , and 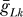 were the same for all candidate models; locations of *K_p_* and *R_Ca_* (middle graph) and 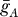 , 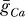 , and 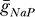 (bottom graph) varied. Some models were shifted slightly along the 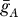 axis to aid in visualization. Subspaces A, C, E, and F were obtained by fixing *K_p_* and *R_Ca_* at the values shown as red dots in the middle graph, while searching over the lower 3-D subspace.
(C) Sensitivity of spontaneous firing rate to *R_Ca_* for candidate models across passive dendrite conductance space. This subpanel is analogous to (A); arrows indicate similar global trends.(1.3 MB TIF)

Figure S6Sensitivity to Morphologic Parameters Depended on the Distributions of Dendritic Ion Channels(A) Sensitivity of spontaneous firing rate to *L* + *SA* and *D* + *SA* with the original distribution of dendritic channels shown in Figure 1E. Top, schematic distribution of dendritic 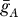 (thick dashed line), 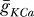 (thin dashed line), 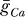 (black solid line), and 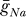 , 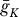 , and 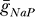 (red solid line). Middle and bottom rows, sensitivities to *L* + *SA* and *D* + *SA* for the candidate models within subspace A, when either dendritic CNs (left column, “CN”) or CDs (right column, “CD”) were held constant. Color represents spontaneous firing rate sensitivities, on the scale at top left (−4 [dark blue] to +4 [dark red]). Arrows indicate the principal sensitivity directions to each parameter perturbation. Sensitivities were positive to CN morphologic perturbations, but negative to CD morphologic perturbations.
(B) Sensitivity of spontaneous firing rate to *L* + *SA* and *D* + *SA* for constant numbers (left column) and constant densities (right column) when all channels have a low, uniform distribution throughout the dendrite (top). Note the order-of-magnitude reduction in the sensitivity colorscale: (−0.4 [dark blue] to +0.4 [dark red]). Sensitivities to all morphologic perturbations were negative. Arrows show the principal sensitivity directions to *L* + *SA* / *CD* and *D* + *SA* / *CN* (top right and bottom left, respectively).(908 KB TIF)

Figure S7Sensitivity of Firing Rate Gain Across Conductance Space for the Passive Dendrite Model(A) Firing rate gain was uniformly low for passive dendrite models within subspace A (colorscale, top right).(B) Firing rate versus injected current for Models 1 (black) and 2 (red) shown in (A). Gain decreased slightly as 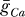 increased.
(C) Gain sensitivities to active parameters *R_Ca_* and 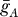 (top row, left and right) and morphologic parameters *D* + *SA* and *L* + *D* (bottom row, left and right) for candidate models in subspace A, according to colorscale at right. Arrows indicate principal sensitivity directions.
(1.6 MB TIF)

Figure S8 Using the Fitness Function to Compare Model and Target Data (A) The AP shape error *E_AP_*. Left: A window W (gray) was centered around each model (solid red line) and target AP (dashed black line). Right: After shifting the model window along the time axis, the root mean squared difference between the model and target traces was computed.
(B) Instantaneous firing rates versus time of model (filled red triangles, solid red line) and target data (filled black squares, dashed line) in response to a 100 pA injection. Lines indicate the best fit of firing rate versus time for each dataset. Gray shading represents *E_FR(Area)_*, the area between firing rate curves, computed at each of the target AP times (open symbols).
(C) Examples of three optimized models (solid red lines) fit to the target data (dashed line). Models were constrained to match the general AP shape, and approximate firing rates, of the synthetic target data. These models show the extremes of the fitness range consistent with firing properties in Area II that were used for the sensitivity analysis.
(970 KB TIF)

Protocol S1 Computing Model Fitness (34 KB DOC)

Table S1 Parameter Values Used to Generate the Target Data These parameters, used in the mathematical model of Equations 1–3, generated the single compartment model output “target data” against which the optimized compartment models were fitted.
(23 KB DOC)

## Abbreviations

AP: action potential
AHP: afterhyperpolarization
AX: axial resistance
Ca^2+^: calcium
CD: constant channel density
CN: constant channel numbers
ISI: interspike interval
K–Ca: Ca^2+^-dependent potassium
