# Supplementary material for: Neuronal Firing Sensitivity to Morphologic and Active Membrane Parameters
Source: PLoS Comput Biol. 2008 Jan 18;4(1):e11. doi: 10.1371/journal.pcbi.0040011 (PMC2211531; doi:10.1371/journal.pcbi.0040011)
Supplement: Supplementary file 5 [file pcbi.0040011.sd005.doc]

**Supporting Protocol S1.	Computing model fitness.**

The fitness *F* of a model *M* to the target data was calculated from the following equation:

$F(M)=\sum_{p=1}^{P} \left(\alpha E_{AP,p}+\beta E_{FR(Slope),p}+\gamma E_{FR(Area),p}+E_{Gen,p}\right)$,	(P1)

where the summation over *p* represents a sum of this fitness function over two current injection protocols [0, 100 pA]. The three error terms *E_AP,p_*, *E_FR(Slope),p_* and *E_FR(Area),p_* were the error in AP shape [42], firing rate area, and firing rate slope between model and target data. The general error term

$E_{Gen,p}=s_{1}E_{Burst,p}+s_{2}E_{Delay,p}+s_{3}E_{Silent,p}$ (P2)

improved optimization performance by penalizing bursting, firing delay and silent models. Appropriate weights for each term in Equations P1 (α, β, γ) and P2 (*s_i_*, *i*=1,..,3) were chosen heuristically and are given below. The computation of each error term for a given protocol *p* is described briefly below.

Action potential shape error, *E_AP_*

The average shape error *E_AP_* was calculated from the individual shape errors *E_AP_(i)* between the *i*^th^ target and model APs within a time window *W*. The *i*^th^ target AP and the *i*^th^ model AP were shifted in time to align their peaks, and the root mean squared (RMS) difference between them, within the window *W*, was calculated (Fig. S8A). The total AP shape error *E_AP_* was the mean of these individual shape errors. We used *W* = [-5,45] ms relative to each AP, and α=2 in Eq. P1. When there were more model than target APs, the shape of each extra model AP was compared against the last target AP.

Firing rate slope error, *E_FR(Slope)_*

The instantaneous firing rate, defined as the reciprocal of each interspike interval, was computed between successive spikes of all model and target data. Fitting a straight line to the instantaneous firing rates versus time, *FR(t) = mt + b*, for the model and target data separately gave slopes *m_M_* and *m_T_* and intercepts *b_M_* and *b_T_* for model and target data respectively (Fig. S8B). Then

$E_{FR(Slope)}=\sqrt{(m_{M}-m_{T})^{2}}$,	(P3)

with the weight β=1000 in Eq. P1.

Firing rate area error, *E_FR(Area)_*

The area between the model and target firing rate versus time curves was computed using the best fit lines defined by (*b_M_*, *m_M_*) and (*b_T_*, *m_T_*) above. Let {*t_i_* , *i*=1,..., *N_T_*} be the times of the target APs. Taking the RMS difference between the model and target lines over each of these times gave

$E_{FR(Area)}=\sqrt{\sum_{i=1}^{N_{T}} \left[(m_{T}(t_{i})-b_{T})-(m_{M}(t_{i})+b_{M})\right]^{2}}$,	(P4)

using the weight γ=0.3 in Eq. P1 above. This error is represented by the shading in Figure S8B.

Burst penalty, *E_Burst_*

The total number of interspike intervals shorter than a user-defined minimum *B* ms. Here, *B*=20, *s_1_* =10.

Firing delay penalty, *E_Delay_*

This function penalized the difference between the timing of the first target and first model AP. The penalty was defined by

$E_{Delay}=\frac{(T_{1}-M_{1})^{2}}{ISI_{T}^{2}}$,	(P5)

where *T_1_* was the time of first target AP, *M_1_* the time of the first model AP, and *ISI_T_* the mean interspike interval of the target data. The parameter *s_2_* = 0.3.

Silent slope penalty, *E_Silent_*

An additional penalty, proportional to the area between firing rate curves defined above, was applied to any model that does not fire any APs:

$E_{Silent}=E_{FR(Area)}$,	(P6)

with *s_3_* = 2.
