# Supplementary material for: Neuronal Firing Sensitivity to Morphologic and Active Membrane Parameters
Source: PLoS Comput Biol. 2008 Jan 18;4(1):e11. doi: 10.1371/journal.pcbi.0040011 (PMC2211531; doi:10.1371/journal.pcbi.0040011)
Supplement: Supplementary file 6 [file pcbi.0040011.st001.doc]

**Supporting Table S1. Parameter values used to generate the target data.** These parameters, used in the mathematical model of Equations (1)-(3), generated the single compartment model output ‘target data’ against which the optimized compartment models were fitted.

| Ion Channel | $\bar{g}$ (mS/cm^2^) | Passive Parameters | |
| --- | --- | --- | --- |
| Na | 10 | $\bar{g}_{Lk}$ (mS/cm^2^) | 0.3 |
| K | 4 | C_m_ (μF/cm^2^) | 1 |
| A | 0 | radius (μm) | 15.5 |
| Ca | 0.25 | Calcium Parameters | |
| K-Ca | 1 | *K_p_* (M-cm^2^/mC) | 0.05 |
| NaP | 0.05 | *R* (ms^-1^) | 0.0125 |
